# COVID-19 hospital admissions and deaths after BNT162b2 and ChAdOx1 nCoV-19 vaccinations in 2·57 million people in Scotland (EAVE II): a prospective cohort study

**DOI:** 10.1016/S2213-2600(21)00380-5

**Published:** 2021-12

**Authors:** Utkarsh Agrawal, Srinivasa Vittal Katikireddi, Colin McCowan, Rachel H Mulholland, Amaya Azcoaga-Lorenzo, Sarah Amele, Adeniyi Francis Fagbamigbe, Eleftheria Vasileiou, Zoe Grange, Ting Shi, Steven Kerr, Emily Moore, Josephine L K Murray, Syed Ahmar Shah, Lewis Ritchie, Dermot O'Reilly, Sarah J Stock, Jillian Beggs, Antony Chuter, Fatemah Torabi, Ashley Akbari, Stuart Bedston, Jim McMenamin, Rachael Wood, Ruby S M Tang, Simon de Lusignan, F D Richard Hobbs, Mark Woolhouse, Colin R Simpson, Chris Robertson, Aziz Sheikh

**Affiliations:** a School of Medicine, University of St Andrews, St Andrews, UK; b MRC/CSO Social & Public Health Sciences Unit, University of Glasgow, Glasgow, UK; c Usher Institute, The University of Edinburgh, Edinburgh, UK; d Public Health Scotland, Glasgow, UK; e Academic Primary Care, University of Aberdeen School of Medicine and Dentistry, Aberdeen, UK; f School of Medicine, Dentistry and Biomedical Sciences, Queen's University Belfast, UK; g BREATHE—The Health Data Research Hub for Respiratory Health, Edinburgh, UK; h Population Data Science, Swansea University Medical School, Swansea, UK; i Nuffield Department of Primary Care Health Sciences, University of Oxford, Oxford, UK; j School of Health, Wellington Faculty of Health, Victoria University of Wellington, Wellington, New Zealand; k Department of Mathematics and Statistics, University of Strathclyde, Glasgow, UK

## Abstract

**Background:**

The UK COVID-19 vaccination programme has prioritised vaccination of those at the highest risk of COVID-19 mortality and hospitalisation. The programme was rolled out in Scotland during winter 2020–21, when SARS-CoV-2 infection rates were at their highest since the pandemic started, despite social distancing measures being in place. We aimed to estimate the frequency of COVID-19 hospitalisation or death in people who received at least one vaccine dose and characterise these individuals.

**Methods:**

We conducted a prospective cohort study using the Early Pandemic Evaluation and Enhanced Surveillance of COVID-19 (EAVE II) national surveillance platform, which contained linked vaccination, primary care, RT-PCR testing, hospitalisation, and mortality records for 5·4 million people (around 99% of the population) in Scotland. Individuals were followed up from receiving their first dose of the BNT162b2 (Pfizer–BioNTech) or ChAdOx1 nCoV-19 (Oxford–AstraZeneca) COVID-19 vaccines until admission to hospital for COVID-19, death, or the end of the study period on April 18, 2021. We used a time-dependent Poisson regression model to estimate rate ratios (RRs) for demographic and clinical factors associated with COVID-19 hospitalisation or death 14 days or more after the first vaccine dose, stratified by vaccine type.

**Findings:**

Between Dec 8, 2020, and April 18, 2021, 2 572 008 individuals received their first dose of vaccine—841 090 (32·7%) received BNT162b2 and 1 730 918 (67·3%) received ChAdOx1. 1196 (<0·1%) individuals were admitted to hospital or died due to COVID-19 illness (883 hospitalised, of whom 228 died, and 313 who died due to COVID-19 without hospitalisation) 14 days or more after their first vaccine dose. These severe COVID-19 outcomes were associated with older age (≥80 years *vs* 18–64 years adjusted RR 4·75, 95% CI 3·85–5·87), comorbidities (five or more risk groups *vs* less than five risk groups 4·24, 3·34–5·39), hospitalisation in the previous 4 weeks (3·00, 2·47–3·65), high-risk occupations (ten or more previous COVID-19 tests *vs* less than ten previous COVID-19 tests 2·14, 1·62–2·81), care home residence (1·63, 1·32–2·02), socioeconomic deprivation (most deprived quintile *vs* least deprived quintile 1·57, 1·30–1·90), being male (1·27, 1·13–1·43), and being an ex-smoker (ex-smoker *vs* non-smoker 1·18, 1·01–1·38). A history of COVID-19 before vaccination was protective (0·40, 0·29–0·54).

**Interpretation:**

COVID-19 hospitalisations and deaths were uncommon 14 days or more after the first vaccine dose in this national analysis in the context of a high background incidence of SARS-CoV-2 infection and with extensive social distancing measures in place. Sociodemographic and clinical features known to increase the risk of severe disease in unvaccinated populations were also associated with severe outcomes in people receiving their first dose of vaccine and could help inform case management and future vaccine policy formulation.

**Funding:**

UK Research and Innovation (Medical Research Council), Research and Innovation Industrial Strategy Challenge Fund, Scottish Government, and Health Data Research UK.

## Introduction

The COVID-19 pandemic has resulted in over 220 million cases and 4·6 million deaths worldwide as of September, 2021, leading to large-scale disruption of societies. These figures include more than 7·2 million cases and almost 130 500 deaths in the UK.^1^ The emergence and subsequent spread of this novel virus has resulted in devastating global social and economic consequences, affecting nearly every aspect of our lives.^2^ Although public health actions, such as lockdown measures, physical distancing, and improved hygiene practices, have reduced the likelihood of transmission, an urgent need for mass deployment of vaccines against SARS-CoV-2 remains.^3^

The COVID-19 vaccination programme started in Scotland on Dec 8, 2020, beginning with the BNT162b2 (Pfizer–BioNTech) COVID-19 vaccine. After approval, the ChAdOx1 nCoV-19 (Oxford–AstraZeneca) vaccine was also deployed in the Scottish vaccination programme from Jan 4, 2021. A two-dose schedule is advised for both of these vaccines in the UK.^4^ During winter 2020–21, the pandemic in Scotland resulted in the highest incidence of SARS-CoV-2 recorded since the start of the pandemic in March, 2020, and until the end of May, 2021. This winter peak began around the first week of December, 2020, peaked on Jan 4, 2021, and continued to decline after that, which coincided with the period of vaccination rollout reported in this study.^5^

**Research in context**

**Evidence before this study**

We searched PubMed, medRxiv, and SSRN on May 27, 2021, for studies using free text and related MeSH terms for hospital admission following COVID-19 vaccination, using the terms “COVID-19 breakthrough infections”, “COVID-19 vaccines (MeSH)”, and “COVID-19 (MeSH)”. We only considered studies published in English. A study in Israel on 596 618 individuals who received the BNT162b2 (Pfizer–BioNTech) vaccine reported that 110 vaccinated individuals were admitted to hospital and there were nine deaths due to COVID-19 up to 42 days after vaccination. However, nearly all individuals (96%) in the study received a second dose of vaccination (95% of participants received the second dose before day 24). Public Health England reported that 9% of people aged 80 years or older who tested positive for COVID-19 after a first dose of BNT162b2 vaccine and 7% of people who received a first dose of the ChAdOx1 (Oxford–AstraZeneca) vaccine were subsequently admitted to hospital for COVID-19. A preprint study in northwest London followed up 389 587 people for an average of 29 days after the first dose of vaccine, with 288 patients admitted to hospital (155 in the first 14 days after vaccination). A separate report of the ISARIC/CO-CIN study in the UK reported that, since the start of the vaccination programme, one in 25 people admitted to hospital for COVID-19 had received at least one vaccine dose. Of the 42 788 people recruited to this study since Dec 8, 2020, 4·2% had been vaccinated (1685 with one dose and 27 with two doses), with a median time from vaccination to admission to hospital of 10 days.

**Added value of this study**

This national-level analysis found that at 14 days or longer since the first vaccine dose, there were 883 COVID-19 admissions to hospital and 541 deaths in almost 2·57 million individuals in Scotland. Older age, increasing number of underlying comorbidities, recent admission to hospital, being in a high-risk occupation, being a care home resident, being male, being socioeconomically deprived, and being an ex-smoker were all associated with an increased risk of severe post-vaccine COVID-19 events. By contrast, previous COVID-19 infection had a protective effect.

**Implications of all the available evidence**

The rollout of the COVID-19 vaccination programme was associated with low numbers of post-vaccination serious COVID-19 outcomes at 14 days or longer since vaccination. Whether the same degree of protection is maintained beyond the analysed period of follow-up and once lockdown measures are lifted remains to be seen. As the rollout of vaccination programmes continues worldwide, there is a need to identify those at increased risk of breakthrough infections and identify mechanisms to reduce those risks. The benefits of an expedited second dose compared with a longer gap between vaccinations are unclear, as there is potential longer-term gain from an enhanced immune response with a longer interval between the doses.

The COVID-19 vaccination programme in Scotland has adhered to advice from the UK Joint Committee on Vaccination and Immunisation (JCVI).^6^ The JCVI initially advised that, in the context of restricted vaccine supplies, an offer of a first dose followed by a second dose 12 weeks later was desirable to provide at least some protection for as many people as soon as possible. The JCVI prioritised vaccinations for those at highest risk of COVID-19 mortality (appendix pp 1–3). Thus, the vaccine rollout began with care home residents, health and care workers, and people aged 80 years and older, followed by combinations of clinically vulnerable people and progressively younger age groups. Although this advice differs from trial evidence and manufacturers guidance on timing between doses,^7^, ^8^, ^9^ data from mathematical models suggest that this approach has the potential to contain the pandemic quickly, achieving optimal public health benefit,^10^ and could result in reduced cumulative mortality under certain conditions.^11^ Additionally, given that vaccine demand will probably exceed supply for some time, several countries around the world have either adopted the strategy of delaying provision of a second dose in their programmes or are considering doing so.^12^, ^13^

Clinical trials for the licenced vaccines showed 95% efficacy for BNT162b2 and 70% efficacy for ChAdOx1 against symptomatic disease after two vaccine doses.^7^, ^14^, ^15^ However, these studies might not necessarily reflect real-world outcomes because of exclusion or under-representation of certain populations (such as older age groups and those with comorbidities), differences in risk of infection during the observation periods, different criteria for diagnosis and grading the severity of infection between a trial and routine clinical practice, and the appearance of new SARS-CoV-2 variants.^16^, ^17^ Analysis of real-world data from the vaccination programme in Scotland between Dec 8, 2020, and Feb 22, 2021, found that the first dose of the mass vaccination programme was associated with a 91% (95% CI 85–94) reduction in COVID-19 admissions to hospital for the BNT162b2 vaccine and an 88% (75–94) reduction for ChAdOx1 at 28–34 days post-vaccination.^18^

Even with highly effective vaccines, some vaccinated individuals will still become infected with SARS-CoV-2 and develop severe COVID-19.^19^ It is important to understand the frequency of such severe COVID-19 outcomes and identify who is at the greatest risk to inform clinical practice, public health strategy, and evolving vaccination policy. We sought to estimate the frequency of COVID-19 admissions to hospital or deaths 14 days or more after receiving the first vaccine dose and to characterise individuals with these outcomes in terms of demographic and clinical considerations.

## Methods

### Study design and population

The Early Pandemic Evaluation and Enhanced Surveillance of COVID-19 (EAVE II) platform is an open, real-time, prospective observational cohort with national-level coverage in Scotland that uses linked vaccination, primary care, laboratory testing, hospitalisation, and mortality data, and has been described in detail elsewhere.^20^ Data were available for 5·4 million people in Scotland (around 99% of the population) within the EAVE II platform. The population of interest was all adults (aged ≥18 years on Dec 8, 2020) who received the first dose of either the BNT162b2 vaccine or the ChAdOx1 vaccine by April 18, 2021, and who did not have a COVID-19 hospitalisation or any death less than 14 days after vaccination. Events less than 14 days after vaccination were excluded to allow time for an immune response to develop and to avoid inclusion of any infections acquired before vaccination.^21^

Data on all individuals from 940 general practices across Scotland were deterministically linked to data on SARS-CoV-2 testing, the vaccination programme, hospital admissions, and mortality records using the Community Health Index number, which is a unique identifier used in all health-care contacts across Scotland. Data were available for each individual from general practice (GP) records, the Electronic Communication of Surveillance in Scotland (ECOSS) for laboratory data, vaccination data from GP records and the Turas Vaccination Management Tool (TVMT),^22^ the Scottish Morbidity Record database and Rapid Preliminary Inpatient Data for hospital admission data,^23^ and National Records of Scotland death registry for mortality data. Laboratory data from ECOSS included all RT-PCR test results from both UK National Health Service (NHS) laboratories (pillar 1) and Lighthouse Government laboratories (pillar 2).^24^ Vaccination information was available on individuals vaccinated in general practices directly from GP records. Information on those vaccinated in community vaccination hubs and any other settings, such as care homes and hospitals, was obtained from the national TVMT database.^22^

Our population of interest included individuals for whom 14 days or more had passed after receiving a first dose of vaccine between Dec 8, 2020, and April 18, 2021, until admission to hospital for COVID-19, death due to COVID-19, or the end of the study on April 18, 2021. Vaccinated groups were stratified by time intervals for 14–20 days, 21–27 days, 28–34 days, 35–41 days, and 42 or more days post-vaccination, and by the type of vaccine received.

Ethical approval was granted by the National Research Ethics Service Committee, Southeast Scotland 02 (reference number 12/SS/0201). Approval for data linkage and its use was granted by the Public Benefit and Privacy Panel for Health and Social Care (reference number 1920-0279) and hence individual written patient consent was not required.

### Outcomes

We identified COVID-19 hospital admissions that occurred between Dec 22, 2020 (14 days after the vaccination programme started), and April 18, 2021. Cases were defined as an individual admitted to hospital with COVID-19 as the recorded cause of admission, those with any hospital admissions within 14 days of a positive RT-PCR test for SARS-CoV-2 infection, or those with any admission to hospital for a non-COVID-19 reason and with a positive RT-PCR test for SARS-CoV-2 infection during that admission (appendix p 4).

We defined COVID-19 deaths as any individual with COVID-19 as the main International Classification of Diseases (ICD)-10 cause of death recorded on the death certificate, or death from any cause within 28 days of a positive RT-PCR test for SARS-CoV-2 infection (appendix p 4).^1^

### Population characteristics and covariates

We identified characteristics of interest at baseline for the study population on Dec 8, 2020, namely age, sex, socioeconomic status measured by quintiles of the Scottish Index of Multiple Deprivation (SIMD), residential settlement measured by the urban rural six-fold classification, health board of residence, COVID-19 before vaccination, smoking status, the number and types of comorbidities commonly associated with severe COVID-19 illness, and calendar week of vaccination. We identified people living in households of more than ten people where the average age of residents was 65 years or older and used this as a proxy for living in a residential care home.

SIMD was allocated based on an individual's home postcode, with quintiles of population ranging from 1 for the most deprived 20% to 5 for the least deprived 20% of the population. Residential settlement is a measure of rurality that ranges from 1 for large urban areas to 6 for small remote rural areas. Comorbidities of interest included a history of asthma, chronic kidney disease, liver cirrhosis, chronic neurological condition, heart failure, diabetes (type 1 and type 2), dementia, or coronary heart disease, since these have been shown to be associated with poorer COVID-19 outcomes.^25^ Smoking status (never smoker, current smoker, or ex-smoker), blood pressure (normal, low, high, very high, no investigation, and unknown), and body-mass index (BMI) were also included as risk factors within the analysis. The number of previous SARS-CoV-2 tests (a proxy for high-risk occupational groups who were repeatedly tested) and previously testing positive were retrieved from laboratory testing data. We also identified people who had been admitted to hospital in the 4 weeks before their first vaccination.

### Statistical analysis

We summarised the characteristics of individuals with severe COVID-19 outcomes of interest (ie, admitted to hospital or died due to COVID-19 illness) 14 days or more after the first dose of vaccine overall and by each vaccine type. Baseline characteristics of the study population were reported for demographic and clinical characteristics of interest using counts and proportions. These factors were also reported by the type of vaccine used and by those who subsequently were admitted to hospital or died because of COVID-19 at least 14 days after vaccination. For people with an event of interest, we calculated the absolute risk per 1000 person-years for each factor based on the total follow-up of individuals within each group of interest.

We used generalised linear models assuming a Poisson distribution with person-time for an offset representing the time at risk to derive rate ratios (RRs) with 95% CIs for admission to hospital or death from COVID-19. We calculated both the unadjusted and adjusted RRs for all predictors including age, sex, previous history of COVID-19, SIMD, urban rural classification, number of risk groups, care home residence, the number of previous SARS-CoV-2 tests (a proxy for high-risk occupational groups), smoking status, previous history of hospitalisation (admission to hospital within 4 weeks before vaccination), and time since first vaccination. RRs for individual comorbidities were adjusted for age, sex, and SIMD in a separate model for each condition. Calendar time intervals by week and registered health board were also included as confounders to account for the evolving state of the pandemic over time and by region. A count of conditions was included in our final adjusted model rather than individual conditions due to multicollinearity between these factors. Survival analysis that took into account the time at risk was used to derive cumulative incidence plots for occurrence of admission to hospital or death due to COVID-19. All statistical tests were two-tailed with a 5% significance level. R (version 3.5.1) was used to carry out all statistical analyses, which were performed by one statistician (UA) and subsequently independently checked by a second statistician (CR or EM).

A statistical analysis plan was developed before undertaking the analysis and published on the EAVE II project website.^26^ RECORD^27^ and STROBE^28^ were used to guide transparent reporting (appendix pp 5–9). Our analysis code will be made publicly available at the time of publication.

### Role of the funding source

The funder of the study had no role in study design, data collection, data analysis, data interpretation, or writing of the report.

## Results

**Figure 1:**
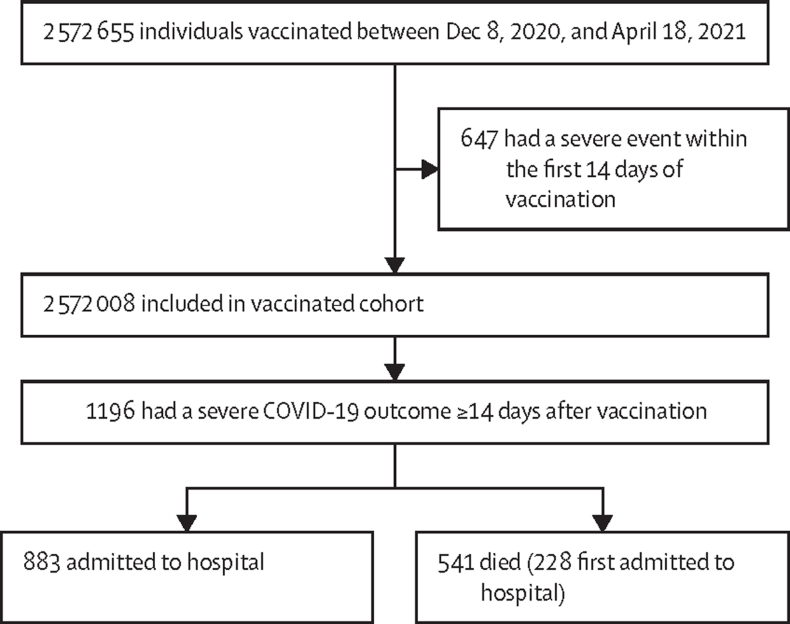
Flow chart representing vaccine coverage in Scotland between Dec 8, 2020, and April 18, 2021, including severe outcomes due to COVID-19 14 days or more after first vaccine dose 313 patients died without being admitted to hospital.

Between Dec 8, 2020, and April 18, 2021, 2 570 812 (>99·9%) of 2 572 008 individuals aged 18 years and older who were administered a first dose of COVID-19 vaccine did not have any severe COVID-19 outcome less than 14 days after vaccination and so were eligible for inclusion in the study (figure 1; appendix pp 10–14). Eligible individuals were followed up for 391 571 person-years until any admission to hospital for COVID-19 illness, COVID-19 death, or the end of follow-up on April 18, 2021 (table 1). The median follow-up after first vaccination was 55 days (IQR 31–67). The range of follow-up after first and second doses of vaccine was to a maximum of 128 days.

**Table 1:** Characteristics of individuals in Scotland with any first vaccination and COVID-19 hospitalisations or deaths (severe COVID-19 outcome) 14 days or more after vaccination

		**Total vaccination (n=2 572 008)**	**Severe COVID-19 outcome, n (rate per 1000 person-years)**
Overall		..	1196 (4·6)
Sex			
	Female	1 418 305 (55·1%)	681 (4·5)
	Male	1 153 703 (44·9%)	515 (4·7)
Age, years			
	18–64	1 659 660 (64·5%)	235 (1·9)
	65–79	697 505 (27·1%)	327 (3·3)
	≥80	214 843 (8·4%)	634 (18·6)
Previous history of COVID-19			
	No	2 488 274 (96·7%)	1154 (4·6)
	Yes	83 734 (3·3%)	42 (5·4)
Previous history of hospitalisation*			
	No	2 511 281 (97·6%)	1068 (4·2)
	Yes	60 727 (2·4%)	128 (20·4)
Residential care home			
	No	2 552 805 (99·3%)	936 (3·6)
	Yes	19 203 (0·7%)	260 (96·5)
Deprivation status			
	1—Most deprived	468 835 (18·2%)	328 (6·9)
	2	504 810 (19·6%)	285 (5·5)
	3	526 056 (20·5%)	194 (3·6)
	4	537 664 (20·9%)	195 (3·6)
	5—Least deprived	521 213 (20·3%)	181 (3·5)
	Unknown	13 430 (0·5%)	13 (10·2)
Urban rural score			
	1—Large urban area	776 050 (30·3%)	365 (4·7)
	2	990 996 (38·7%)	497 (4·9)
	3	258 023 (10·1%)	119 (4·5)
	4	138 885 (5·4%)	52 (3·6)
	5	248 218 (9·7%)	124 (5·0)
	6—Remote rural area	146 406 (5·7%)	26 (1·8)
	Unknown	13 430 (0·5%)	13 (10·2)
Smoking status			
	Ex-smoker	432 792 (16·8%)	312 (6·3)
	Non-smoker	999 523 (38·9%)	391 (3·9)
	Smoker	621 559 (24·2%)	301 (4·7)
	Unknown	518 134 (20·1%)	192 (4·2)
Number of comorbidities†			
	0	1 233 224 (47·9%)	177 (1·6)
	1	760 243 (29·6%)	207 (2·7)
	2	337 957 (13·1%)	258 (6·4)
	3	141 200 (5·5%)	236 (12·7)
	4	60 156 (2·3%)	138 (16·5)
	≥5	39 228 (1·5%)	180 (32·1)
Number of previous COVID-19 tests‡			
	0	2 059 613 (80·1%)	524 (2·6)
	1	313 028 (12·2%)	128 (5·8)
	2	78 230 (3·0%)	110 (12·6)
	3	29 412 (1·1%)	101 (28·2)
	4–9	47 448 (1·8%)	208 (32·9)
	≥10	44 277 (1·7%)	71 (11·6)
Medical history			
	Asthma	352 672 (13·7%)	169 (4·7)
	Chronic kidney disease (stages 3–5)	151 969 (5·9%)	318 (14·4)
	Liver cirrhosis	20 301 (0·8%)	16 (6·8)
	Chronic neurological condition	16 897 (0·7%)	9 (4·7)
	Heart failure	44 663 (1·7%)	94 (15·1)
	Type 1 diabetes	20 325 (0·8%)	8 (3·8)
	Type 2 diabetes	243 918 (9·5%)	288 (9·3)
	Dementia	34 552 (1·3%)	259 (51·8)
	Coronary heart disease	190 015 (7·4%)	278 (10·7)

Data are n (%), unless otherwise indicated. Percentages might not sum to 100 because of rounding.

* Previous history of hospitalisation status defined as admission to hospital within 4 weeks before the first vaccination.

† Individual QCOVID risk groups found in the appendix (pp 21–22).

‡ A proxy for high-risk occupations.

841 090 (32·7%) individuals received a first dose of BNT162b2 and 1 730 918 (67·3%) individuals received a first dose of ChAdOx1 vaccine (table 2). 694 915 (27·0%) individuals received a second vaccine dose within the study period. 412 649 (59·4%) individuals received a second dose of BNT162b2 and 282 266 (40·6%) individuals received a second dose of ChAdOx1 with a combined 43 166 years of follow-up after the second dose. The median follow-up after the second dose was 19 days (IQR 8–33).

**Table 2:** Characteristics of individuals in Scotland and number and rate of severe COVID-19 outcomes 14 days or more after first dose of vaccine

		**BNT162b2 vaccination (n=841 090)**	**Severe COVID-19 outcome among BNT162b2 recipients, n (rate per 1000 person-years)**	**ChAdOx1 vaccination (n=1 730 918)**	**Severe COVID-19 outcome among ChAdOx1 recipients, n (rate per 1000 person-years)**
Overall		..	593 (5·3)	..	603 (4·1)
Sex					
	Female	525 708 (62·5%)	353 (4·9)	892 597 (51·6%)	328 (4·2)
	Male	315 382 (37·5%)	240 (5·8)	838 321 (48·4%)	275 (4·0)
Age, years					
	18–64	476 007 (56·6%)	107 (1·8)	1 183 653 (68·4%)	128 (1·9)
	65–79	328 733 (39·1%)	158 (3·3)	368 772 (21·3%)	169 (3·3)
	≥80	36 350 (4·3%)	328 (62·8)	178 493 (10·3%)	306 (10·6)
Previous history of COVID-19					
	No	807 322 (96·0%)	562 (5·2)	1 680 952 (97·1%)	592 (4·1)
	Yes	33 768 (4·0%)	31 (7·0)	49 966 (2·9%)	11 (3·3)
Previous history of hospitalisation*					
	No	828 156 (98·5%)	552 (5·0)	1 683 125 (97·2%)	516 (3·6)
	Yes	12 934 (1·5%)	41 (25·2)	47 793 (2·8%)	87 (18·7)
Residential care home					
	No	822 585 (97·8%)	336 (3·0)	1 730 220 (>99·9%)	600 (4·1)
	Yes	18 505 (2·2%)	257 (98·3)	698 (<0·1%)	3 (37·3)
Deprivation status					
	1—Most deprived	155 179 (18·4%)	142 (6·8)	313 656 (18·1%)	186 (7·0)
	2	166 798 (19·8%)	131 (5·8)	338 012 (19·5%)	154 (5·3)
	3	168 335 (20·0%)	115 (5·2)	357 721 (20·7%)	79 (2·5)
	4	178 334 (21·2%)	105 (4·4)	359 330 (20·8%)	90 (3·0)
	5—Least deprived	167 512 (19·9%)	88 (3·8)	353 701 (20·4%)	93 (3·2)
	Unknown	4932 (0·6%)	12 (18·8)	8498 (0·5%)	1 (1·6)
Urban rural score					
	1—Large urban area	250 598 (30·0%)	160 (4·7)	525 452 (30·5%)	205 (4·7)
	2	340 101 (40·7%)	240 (5·2)	650 895 (37·8%)	257 (4·7)
	3	81 977 (9·8%)	53 (4·9)	176 046 (10·2%)	66 (4·3)
	4	42 682 (5·1%)	33 (6·0)	96 203 (5·6%)	99 (2·1)
	5	72 339 (8·7%)	80 (8·3)	175 879 (10·2%)	44 (2·9)
	6—Remote rural area	48 461 (5·8%)	15 (2·5)	97 945 (5·7%)	11 (1·2)
	Unknown	4932 (0·6%)	12 (18·8)	8498 (0·5%)	1 (1·6)
Smoking status					
	Ex-smoker	139 457 (16·6%)	139 (7·2)	293 335 (16·9%)	173 (5·6)
	Non-smoker	333 702 (39·7%)	189 (4·2)	665 821 (38·5%)	202 (3·6)
	Smoker	194 897 (23·2%)	129 (4·9)	426 662 (24·6%)	172 (4·6)
	Unknown	173 034 (20·6%)	136 (6·0)	345 100 (19·9%)	56 (2·4)
Number of comorbidities†					
	0	420 033 (49·9%)	82 (1·5)	813 191 (47·0%)	95 (1·8)
	1	244 230 (29·0%)	85 (2·6)	516 013 (29·8%)	122 (2·7)
	2	103 811 (12·3%)	124 (8·8)	234 146 (13·5%)	134 (5·2)
	3	42 751 (5·1%)	124 (20·9)	98 449 (5·7%)	112 (8·9)
	4	18 212 (2·2%)	76 (29·8)	41 944 (2·4%)	62 (10·7)
	≥5	12 053 (1·4%)	102 (59·9)	27 175 (1·6%)	78 (19·9)
Number of previous COVID-19 tests‡					
	0	612 187 (72·8%)	151 (1·9)	1 447 426 (83·6%)	373 (3·0)
	1	114 362 (13·6%)	87 (5·6)	198 666 (11·5%)	95 (6·0)
	2	33 236 (4·0%)	67 (14·7)	44 994 (2·6%)	43 (10·4)
	3	14 665 (1·7%)	82 (40·6)	14 747 (0·9%)	19 (12·1)
	4–9	29 236 (3·5%)	154 (37·2)	18 212 (1·1%)	54 (24·8)
	≥10	37 404 (4·4%)	52 (9·7)	6873 (0·4%)	19 (25·8)
Medical history					
	Asthma	110 799 (13·2%)	57 (5·3%)	241 873 (14·0%)	112 (3·9)
	Chronic kidney disease (stages 3–5)	44 133 (5·2%)	142 (11·1%)	107 836 (6·2%)	176 (22·6)
	Liver cirrhosis	6237 (0·7%)	4 (7·9%)	14 064 (0·8%)	12 (4·7)
	Chronic neurological condition	4567 (0·5%)	6 (2·3%)	12 330 (0·7%)	3 (10·0)
	Heart failure	12 283 (1·5%)	31 (14·0)	32 380 (1·9%)	63 (18·0)
	Diabetes (type 1)	5582 (0·7%)	4 (2·9)	14 743 (0·9%)	4 (5·6)
	Diabetes (type 2)	79 611 (9·5%)	129 (7·9)	164 307 (9·5%)	159 (11·8)
	Dementia	17 274 (2·1%)	225 (13·2)	17 278 (1·0%)	34 (92·6)
	Coronary heart disease	60 379 (7·2%)	120 (9·0)	129 636 (7·5%)	158 (14·2)

Data are n (%), unless otherwise indicated. Percentages might not sum to 100 because of rounding.

* Previous history of hospitalisation status defined as admission to hospital within 4 weeks before first dose of vaccine.

† Individual QCOVID risk groups found in the appendix (pp 21–22).

‡ A proxy for high-risk occupations.

1196 (<0·1%) individuals (4·6 events per 1000 person-years) had the outcome of interest (table 1). 883 individuals were admitted to hospital and 541 people died with COVID-19 14 days or more after the first vaccination, 228 of whom died after hospitalisation (figure 1). There were 39 deaths and hospitalisations among individuals who received a second vaccine dose, which translated into an event rate of 0·9 per 1000 person-years. Among individuals who received BNT162b2 and ChAdOx1 vaccines, there were 593 (5·3 events per 1000 person-years) and 603 (4·1 events per 1000 person-years) events of interest, respectively (table 2). The rate of events differed for the two vaccines most notably in those aged 80 years and older, with people receiving BNT162b2 having a rate of 62·8 events per 1000 person-years compared with a rate of 10·6 per 1000 person-years for ChAdOx1.

**Figure 2:**
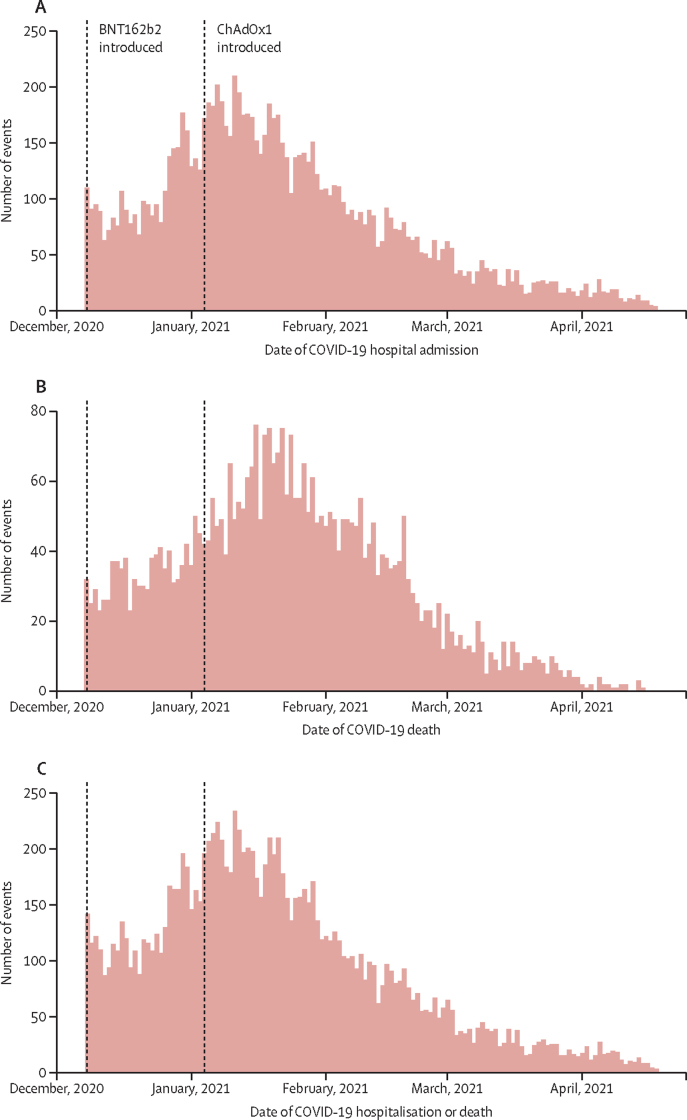
Severe COVID-19 outcomes in Scotland in winter 2020–21 for hospitalisation (A), deaths (B), and hospitalisation and deaths combined (C)

We recorded the distribution of hospitalisations due to COVID-19, deaths due to COVID-19 and these events together in the Scottish population over winter 2020/21 when the study was conducted (figure 2). We also generated the same outcomes stratified by age and vaccine type among vaccinated individuals (appendix pp 23–28).

We reported adjusted RRs for demographic and clinical characteristics and associations with severe COVID-19 outcomes (table 3; unadjusted RRs are shown in the appendix pp 15–17). When considering adjusted data for both vaccines combined, older age, higher number of comorbidities, hospitalisation in the previous 4 weeks before vaccination, having a high-risk occupation, residing in a care home, living in areas with the highest deprivation, being male, and being an ex-smoker increased the risk of hospitalisation or death from COVID-19 (table 3). Previous history of COVID-19 was protective against severe COVID-19 outcomes (table 3).

**Table 3:** Associations for multivariate Poisson models for demographic and clinical characteristics of patients with hospitalisations or death due to COVID-19 14 days or more after first vaccine dose

	**Both vaccines**	**BNT162b2**	**ChAdOx1**
**Time since first vaccination, days**			
14–20	1 (ref)	1 (ref)	1 (ref)
21–27	0·66 (0·56–0·78)	0·73 (0·57–0·94)	0·61 (0·49–0·76)
28–34	0·48 (0·40–0·57)	0·63 (0·49–0·82)	0·37 (0·28–0·48)
35–41	0·37 (0·30–0·45)	0·43 (0·33–0·60)	0·31 (0·24–0·42)
42–128	0·23 (0·20–0·27)	0·33 (0·27–0·42)	0·16 (0·13–0·20)
**Sex**			
Female	1 (ref)	1 (ref)	1 (ref)
Male	1·27 (1·13–1·43)	1·45 (1·22–1·73)	1·11 (0·94–1·31)
**Age, years**			
18–64	1 (ref)	1 (ref)	1 (ref)
65–79	1·95 (1·57–2·42)	3·73 (2·70–5·16)	0·96 (0·71–1·29)
≥80	4·75 (3·85–5·87)	8·26 (6·09–11·21)	1·87 (1·31–2·65)
**Previous history of COVID-19**			
No	1 (ref)	1 (ref)	1 (ref)
Yes	0·40 (0·29–0·54)	0·42 (0·29–0·61)	0·38 (0·21–0·70)
Previous history of hospitalisation*			
No	1 (ref)	1 (ref)	1 (ref)
Yes	3·00 (2·47–3·65)	3·14 (2·26–4·37)	2·74 (2·14–3·50)
**Residential care home**			
No	1 (ref)	1 (ref)	1 (ref)
Yes	1·63 (1·32–2·02)	1·33 (1·06–1·68)	1·36 (0·42–4·39)
**Deprivation status**			
1—Most deprived	1·57 (1·30–1·90)	1·31 (0·99–1·74)	1·83 (1·41–2·38)
2	1·40 (1·16–1·70)	1·27 (0·96–1·68)	1·54 (1·19–2·01)
3	1·00 (0·81–1·22)	1·02 (0·76–1·35)	0·95 (0·70–1·30)
4	1·02 (0·83–1·25)	0·93 (0·70–1·24)	1·13 (0·84–1·51)
5—Least deprived	1 (ref)	1 (ref)	1 (ref)
**Urban rural index**			
1 (Large urban areas, other urban areas, accessible small towns)	1 (ref)	1 (ref)	1 (ref)
2 (Remote small towns, accessible rural areas, remote rural areas)	1·16 (0·97–1·38)	1·15 (0·91–1·46)	1·03 (0·79–1·35)
**Smoking status**			
Non-smoker	1 (ref)	1 (ref)	1·0
Smoker	1·14 (0·97–1·33)	1·30 (1·03–1·65)	1·00 (0·81–1·24)
Ex-smoker	1·18 (1·01–1·38)	1·23 (0·98–1·54)	1·15 (0·93–1·41)
Unknown	1·06 (0·88–1·27)	1·02 (0·81–1·29)	1·15 (0·83–1·58)
**Number of risk groups**†			
0	1 (ref)	1 (ref)	1 (ref)
1	1·39 (1·13–1·71)	1·35 (0·99–1·84)	1·29 (0·97–1·71)
2	2·40 (1·95–2·95)	2·54 (1·86–3·46)	1·91 (1·44–2·53)
3	3·18 (2·55–3·95)	3·11 (2·24–4·33)	2·58 (1·92–3·48)
4	2·90 (2·26–3·73)	2·64 (1·83–3·82)	2·56 (1·81–3·61)
≥5	4·24 (3·34–5·39)	3·70 (2·60–5·26)	3·94 (2·82–5·50)
**Number of previous COVID-19 tests**			
0	1 (ref)	1 (ref)	1 (ref)
1	1·83 (1·54–2·18)	1·91 (1·44–2·54)	1·71 (1·36–2·15)
2	2·21 (1·77–2·77)	2·02 (1·45–2·82)	2·27 (1·64–3·13)
3	2·86 (2·25–3·65)	2·91 (2·10–4·03)	2·09 (1·31–3·34)
4–9	3·01 (2·47–3·67)	2·85 (2·13–3·81)	3·32 (2·45–4·51)
≥10	2·14 (1·62–2·81)	1·92 (1·34–2·75)	3·51 (2·17–5·69)

Data are adjusted (for age, sex, previous history of COVID-19, Scottish Index of Multiple Deprivation, urban–rural classification, number of risk groups, care home residence, number of previous COVID-19 tests [a proxy for high-risk occupational groups], smoking status, previous history of hospitalisation [admission to hospital within 4 weeks before vaccination], and time since first vaccination) RR (95% CI). RR=rate ratio.

* Previous history of hospitalisation status defined as admission to hospital within 4 weeks before first dose of vaccine.

† Individual QCOVID risk groups found in the appendix (pp 21–22).

For BNT162b2, older age, increasing number of comorbidities, living in a care home, having a high-risk occupation, being in the most socially deprived classification, recent admission to hospital, being an ex-smoker, and living in a more rural location were all associated with an increased risk of severe COVID-19 outcomes, whereas previous history of COVID-19 was associated with a reduced risk (table 3). For ChAdOx1, older age, increasing number of comorbidities, having a high-risk occupation, being in the most socially deprived classification, and recent admission to hospital were associated with an increased risk of severe COVID-19 outcomes, whereas previous history of COVID-19 was associated with a reduced risk (table 3).

The 14–20-day period after vaccination had a greater risk of severe COVID-19 outcomes than later periods for the vaccines combined and individually. People who were vaccinated with a history of asthma, chronic kidney condition, heart failure, type 2 diabetes, dementia, and coronary heart disease were at an increased risk of admission to hospital or death due to COVID-19 after adjusting for age, sex, and SIMD (table 4). When considering people vaccinated with BNT162b2 separately, we found no association between asthma or heart failure and an increased risk of adverse COVID-19 outcomes. We reported cumulative incidence plots for admission to hospital or death due to COVID-19 for vaccine types, age groups, number of risk groups, and socioeconomic status (appendix pp 29–33). We did a sensitivity analysis restricting our case definition to those with a confirmed diagnosis of COVID-19 (ICD code U07.1) or with a positive RT-PCR test, which showed similar results (appendix pp 18–20).

**Table 4:** Associations of conditions of interest with a single vaccination and hospitalisations or death due to COVID-19 14 days or more after first vaccine dose adjusted for age, sex, and deprivation status

	**Both vaccines**	**BNT162b2**	**ChAdOx1**
Asthma	1·19 (1·01–1·40)	0·89 (0·67–1·16)	1·53 (1·24–1·87)
Chronic kidney condition (stage 3–5)	1·60 (1·39–1·84)	1·45 (1·18–1·77)	1·83 (1·51–2·21)
Liver cirrhosis	1·49 (0·87–2·36)	0·97 (0·3–2·26)	1·96 (1·04–3·31)
Chronic neurological condition	1·27 (0·58–2·37)	1·85 (0·66–3·99)	0·79 (0·20–2·06)
Heart failure	1·69 (1·35–2·08)	1·13 (0·76–1·61)	2·26 (1·72–2·92)
Diabetes (type 1)	1·26 (0·54–2·44)	1·39 (0·34–3·62)	1·16 (0·36–2·72)
Diabetes (type 2)	1·81 (1·58–2·07)	1·77 (1·45–2·15)	1·89 (1·57–2·26)
Dementia	5·32 (4·57–6·18)	4·28 (3·45–5·31)	1·66 (1·15–2·33)
Coronary heart disease	1·51 (1·31–1·73)	1·34 (1·08–1·64)	1·71 (1·41–2·06)

Data are adjusted rate ratio (95% CI).

## Discussion

We found a low risk of COVID-19 hospitalisations or deaths 14 days or more after the first vaccination dose, with less than 0·05% of individuals receiving at least one vaccination having a subsequent breakthrough event. Older people, those with underlying long-term conditions, those with a recent hospital admission before vaccination, those with a high-risk occupation, care home residents, men, those with high socioeconomic deprivation, and ex-smokers had a greater risk of COVID-19 hospitalisation or death. With the exception of being an ex-smoker, these factors are known to be associated with an increased risk of severe COVID-19 in the unvaccinated population.^25^ By contrast, previous COVID-19 was associated with a reduced risk of a serious COVID-19 outcome after the first vaccine dose.

The rate of hospitalisation or death for COVID-19 related illness during the study period was 4·6 events per 1000 person-years (1196 events in total). Over the same period, we calculated the rate of hospitalisation or death from COVID-19 as 8·57 events per 1000 person-years (10 282 events in total) in the unvaccinated population in Scotland, despite the fact that this unvaccinated group was a much younger population who the JCVI had assessed to be at a substantially lower risk of severe COVID-19 outcomes.^29^

We excluded events that occurred less than 14 days after vaccination to allow time for the vaccine to trigger an immune response; it is unclear whether the available vaccines confer protection during this initial two week period.^30^, ^31^ Some severe COVID-19 events seen in the initial period after vaccination might also have occurred in those with infection before vaccination.^19^, ^31^ The rate of events during this period was 6·73 per 1000 person-years.

Previous studies have reported impressive vaccine effectiveness in preventing severe outcomes due to COVID-19 after one or two doses, with only 110 admissions to hospital and nine deaths 6 weeks after administration of the first dose of vaccine in almost 600 000 people in Israel.^32^ However, it has become clear that the available vaccines do not prevent 100% of infections and a proportion of those infected will progress to have severe COVID-19 outcomes.^19^ The factors identified as being associated with poorer outcomes in this analysis are largely similar to those reported as risk factors for serious COVID-19 outcomes in unvaccinated populations.^33^ Our finding of poorer outcomes associated with increasing numbers of comorbidities is in keeping with findings from a study in Israel, which suggested that vaccine effectiveness might be slightly lower among people with higher numbers of coexisting conditions.^32^ Similarly, evidence from Public Health England that history of COVID-19 illness increases vaccine response is supported by the reduction in risk of severe COVID-19 outcomes for those with history of the disease seen in this study.^34^ Other work in the USA and Italy has shown that a single mRNA vaccine dose (BNT162b2 or mRNA-1273 [Moderna]) in people with previous COVID-19 elicited similar immune responses to two vaccine doses in patients with no previous history.^35^, ^36^, ^37^

To our knowledge, this is the first national study to estimate and characterise the risk of severe COVID-19 events after the first vaccine dose and the study has several key strengths. First, we used a national platform of relevant linked data based on the Scottish Government's mandated reporting from all NHS providers delivering rapid access to and analysis of data on vaccination status and clinical outcomes from routinely collected electronic health records.^18^, ^20^ Second, the study is population-based, and thus has fewer biases than studies based on selected samples of the population. Third, almost 2·57 million people were included in the analysis, meaning the study was well powered to assess the outcomes of interest overall and by individual vaccine. Therefore, our findings are likely to be relevant across the UK and other countries that are running vaccination programmes using the same vaccines.

A limitation of this study is that we were unable to report on risk of SARS-CoV-2 infection and less severe COVID-19 outcomes. We were limited in this respect by insufficient systematic testing for presence of COVID-19 in the population of those who were vaccinated. We were also unable to make robust comparisons between the vaccines because of differential use of the two deployed vaccines during the study period. From Dec 8, 2020, the first UK-approved vaccine BNT162b2 was targeted towards care home residents, nursing staff, social carers, and frontline health and social care workers. From Jan 4, 2021, onwards, ChAdOx1 became available and was deployed for all prioritised groups, particularly in community settings, because of less onerous storage requirements. During winter 2020–21 the highest incidence in Scotland of SARS-CoV-2 infection at any time during the pandemic was recorded; this began around the first week of December, 2020, peaked on Jan 4, 2021, and continued its decline after that. As vaccine rollouts were started at different times, there were also different background levels of infection for vaccine recipients. We used relatively broad criteria to identify COVID-19 admission and so might have included people who were admitted to hospital or died with COVID-19 but not directly because of it. We performed a sensitivity analysis restricting our case definition to those with a confirmed diagnosis of COVID-19 (ICD code U07.1) or with a positive RT-PCR test. This analysis showed very similar results (appendix pp 18–20). We also examined death records and 516 (95%) of the reported deaths had a confirmed diagnosis of COVID-19 as the main or a contributory cause of death. Although there was no national direction of changes in COVID-19 hospital admission policy over the relatively short duration of this study, there remains the possibility that some of these admissions to hospital were due to a change in admission threshold for COVID-19.

The UK vaccination programme decision to delay administration of second doses to 8–12 weeks rather than the shorter 3-week schedule used in the trials^7^, ^15^ was not based on efficacy data, but rather on trying to maximise coverage with a first dose as quickly as possible.^6^, ^38^ The initial focus was on individuals at highest risk and frontline NHS and care home workers. Our data showing benefit from a single dose and among those at most risk of serious complications is therefore particularly valuable. Other work has also suggested that a longer interval between doses might ultimately trigger a stronger immune response, but this is still an area of uncertainty.^39^, ^40^, ^41^ Monitoring the number of COVID-19-related serious outcomes in the vaccinated population between vaccine doses is important to enable evaluation of the effectiveness of the mass vaccination programme. Such information can help inform future decisions on vaccine schedules, both within the UK and internationally. The use of robust epidemiological studies using routinely collected observational data is vital in assessing real-life effects of the COVID-19 vaccines and vaccination programme. Our analysis was performed during a period where there was a high background incidence of SARS-CoV-2 infection and a national lockdown in place in Scotland (and across the UK), so these findings must be interpreted within this context. We plan to continue analysis as the UK relaxes lockdown restrictions, extends the vaccination programme to younger and healthier individuals, and introduces Moderna and other vaccines into its national vaccination programme.

In conclusion, our findings from this national evaluation of first dose mass vaccination rollout across Scotland found a small risk of hospitalisation or death due to COVID-19 illness 14 days or more days after the first vaccine dose, with older people, those with greater numbers of long-term conditions, people admitted to hospital in the recent weeks before their vaccination, people in high-risk occupations, care home residents, those from deprived backgrounds, men, and ex-smokers at the highest risk. By contrast, previous infection with COVID-19 was associated with a reduced risk of these events following vaccination. Overall, the rate of severe COVID-19 outcomes for individuals from 14 days onwards after a first dose of ChAdOx1 or BNT162b2 was very low, with less than 0·05% of people who received at least one vaccine suffering an adverse breakthrough event.

## Data sharing

A data dictionary covering the data sources used in this study can be found at https://github.com/EAVE-II/EAVE-II-data-dictionary. All code used in this study is publicly available at https://github.com/EAVE-II/Covid-vaccine-failures. The data used in this study are sensitive and will not be made publicly available.

## Declaration of interests

AS, JM, and CR are members of the Scottish Government Chief Medical Officer's COVID-19 Advisory Group. JM is a member of the New and Emerging Respiratory Virus Threats Advisory Group (NERVTAG) and AS is a member of the NERVTAG Risk Stratification Subgroup and an unfunded member of Astra-Zeneca's COVID-19 Strategic Consultancy Group: Thrombocytopenia Taskforce. JM is a member of the Scientific Advisory Group on Emergencies (SAGE) and chairs the COVID Scottish National Incident Management Team and the Scientific Committee of the European Centre for Disease Prevention and Control/WHO-funded IMOVE-COVID-19 group. CM reports research funding from Medical Research Council (MRC), Health Data Research UK, National Institute for Health Research (NIHR), and Scottish Chief Scientist Office (CSO). SJS reports research funding from Wellcome Trust, MRC, NIHR, and Scottish CSO. CRS declares funding from the MRC, NIHR, Scottish CSO, and the New Zealand Ministry for Business, Innovation and Employment and Health Research Council during the conduct of this study. SVK is co-chair of the Scottish Government's Expert Reference Group on COVID-19 and ethnicity, is a member of the SAGE subgroup on ethnicity, and acknowledges funding from a NHS Research Scotland Senior Clinical Fellowship, MRC, and Scottish CSO. CR is a member of the Scientific Pandemic Influenza Group on Modelling and the Medicines and Healthcare Products Regulatory Agency Vaccine Benefit and Risk Working Group. JLKM is a member of the COVID Scottish National Incident Management Team. SdL has received funding through his University from AstraZeneca. FDRH acknowledges part support from the NIHR Applied Research Collaboration Oxford Thames Valley and the NIHR Oxford University Hospital Biomedical Research Centre. All other authors declare no competing interests.
