# Supplementary material for: COVID-19 hospital admissions and deaths after BNT162b2 and ChAdOx1 nCoV-19 vaccinations in 2·57 million people in Scotland (EAVE II): a prospective cohort study
Source: Lancet Respir Med. 2021 Dec;9(12):1439–49. doi: 10.1016/S2213-2600(21)00380-5 (PMC8480963; doi:10.1016/S2213-2600(21)00380-5)
Supplement: Supplementary appendix [file mmc1.pdf]

THE LANCET
Respiratory Medicine

Supplementary appendix

This appendix formed part of the original submission and has been peer reviewed. We post it as supplied by the authors.

Supplement to: Agrawal U, Katikireddi SV, McCowan C, et al. COVID-19 hospital admissions and deaths after BNT162b2 and ChAdOx1 nCoV-19 vaccinations in 2·57 million people in Scotland (EAVE II): a prospective cohort study. *Lancet Respir Med* 2021; published online Sept 29. https://doi.org/10.1016/S2213-2600(21)00380-5.

Supplemental File S1: Context of vaccine roll-out in Scotland

Supplemental Table 1. Joint Committee on Vaccination and Immunisation (JCVI) COVID-19 vaccination priority list on 30 December 2020

Order of priority	Group*	Date rollout of vaccine commenced
1	Residents in a care home for older adults and their carers	8th December 2020
2	All those 80 years of age and over and frontline health and social care workers	
3	All those 75 years of age and over	
4	All those 70 years of age and over and clinically extremely vulnerable individuals	
5	All those 65 years of age and over	
6	All individuals aged 16 years to 64 years with underlying health conditions which put them at higher risk of serious disease and mortality	
7	All those 60 years of age and over	
8	All those 55 years of age and over	
9	All those 50 years of age and over	

*These groups represent around 99% of preventable mortality from COVID-19<br><br>The vaccine roll-out strategy has been determined by an independent UK-wide body, namely the Joint Commission on Vaccinations and Immunisation (JCVI),[1] which has prioritised vaccinations to adults on the basis of assessing the risk of serious COVID-19 outcomes, in particular hospitalisations and deaths.[1]<br><br>Individuals in these priority groups received a written invitation ~14 days before their appointment. They were asked however to delay their vaccination if they had recently had COVID-19, tested positive or were self-isolating. These invitations were accompanied by written advice on the need to observe behavioural measures to reduce the risk of contracting the infection.<br><br>Prior to vaccination, checks were made by the trained administering staff to see if individuals had COVID-19 or tested positive in the preceding 4 weeks; if so, the vaccination was deferred. Immediately following vaccination, individuals received both verbal and written advice on the need to maintain behavioural measures, particularly in the 2-3 weeks following vaccination.<br><br>Because of the different storage requirements for the two vaccines, GPs have administered the Oxford-AstraZeneca vaccine and vaccine centres have mainly administered the Pfizer-BioNTech vaccine. Guided by JCVI priorities, GPs began by focusing their efforts on: a) the mobile elderly who they vaccinated in their general practice surgeries; and b) care home residents affiliated with general practices. Vaccination centres began with focusing on health and social care providers before extending to other	

JCVI priority groups. By February 22nd 2021, Group 7 vaccination was underway and a full roll-out of invitations to Group 6 (Supplemental Table 1) started.<br><br>[7] Joint Committee on Vaccination and Immunisation. Priority groups for coronavirus (COVID-19) vaccination: advice from the JCVI, 30 December 2020. Available from: https://www.gov.uk/government/publications/priority-groups-for-coronavirus-covid-19-vaccination-advice-from-the-jcvi-30-december-2020	

Table 2: ICD-10 codes for COVID-19 illness

Code	Description
U07.1	COVID-19, virus identified
U07.2	COVID-19, virus not identified
B34.2	Coronavirus infection, unspecified site
B97.2	Coronavirus as the cause of diseases classified to other chapters
Source: https://www.isdscotland.org/Products-and-Services/Terminology-Services/docs/COVID-19-Analytical-Guidance-V1-2.pdf	

Table 3: STROBE and RECORD checklists

	Item No.	STROBE items	RECORD items	Location in manuscript where items are reported
**Title and abstract**				
	1	(a) Indicate the study's design with a commonly used term in the title or the abstract (b) Provide in the abstract an informative and balanced summary of what was done and what was found	RECORD 1.1: The type of data used should be specified in the title or abstract. When possible, the name of the databases used should be included.<br>RECORD 1.2: If applicable, the geographic region and timeframe within which the study took place should be reported in the title or abstract.<br>RECORD 1.3: If linkage between databases was conducted for the study, this should be clearly stated in the title or abstract.	p. 1
**Introduction**				
Background rationale	2	Explain the scientific background and rationale for the investigation being reported		p. 1-3
Objectives	3	State specific objectives, including any prespecified hypotheses		p. 1-3
**Methods**				
Study Design	4	Present key elements of study design early in the paper		p. 3
Setting	5	Describe the setting, locations, and relevant dates, including periods of recruitment, exposure, follow-up, and data collection		p. 3-5
Participants	6	*(a) Cohort study* - Give the eligibility criteria, and the sources and methods of selection of participants. Describe methods of follow-up<br>*Case-control study* - Give the eligibility criteria, and the sources and methods of case ascertainment and control selection. Give the rationale for the choice of cases and controls	RECORD 6.1: The methods of study population selection (such as codes or algorithms used to identify subjects) should be listed in detail. If this is not possible, an explanation should be provided.<br><br>RECORD 6.2: Any validation studies of the codes or algorithms used to select the population should be referenced. If validation was conducted for this study and	p. 3-5

		Cross-sectional study - Give the eligibility criteria, and the sources and methods of selection of participants<br><br>*(b) Cohort study* - For matched studies, give matching criteria and number of exposed and unexposed<br>*Case-control study* - For matched studies, give matching criteria and the number of controls per case	not published elsewhere, detailed methods and results should be provided.<br><br>RECORD 6.3: If the study involved linkage of databases, consider use of a flow diagram or other graphical display to demonstrate the data linkage process, including the number of individuals with linked data at each stage.	
Variables	7	Clearly define all outcomes, exposures, predictors, potential confounders, and effect modifiers. Give diagnostic criteria, if applicable.	RECORD 7.1: A complete list of codes and algorithms used to classify exposures, outcomes, confounders, and effect modifiers should be provided. If these cannot be reported, an explanation should be provided.	p.3-5
Data sources/ measurement	8	For each variable of interest, give sources of data and details of methods of assessment (measurement).<br>Describe comparability of assessment methods if there is more than one group		p. 3-4
Bias	9	Describe any efforts to address potential sources of bias		p. 4-5
Study size	10	Explain how the study size was arrived at		N/A
Quantitative variables	11	Explain how quantitative variables were handled in the analyses. If applicable, describe which groupings were chosen, and why		p. 3-5
Statistical methods	12	(a) Describe all statistical methods, including those used to control for confounding<br>(b) Describe any methods used to examine subgroups and interactions<br>(c) Explain how missing data were addressed<br>(d) *Cohort study* - If applicable, explain how loss to follow-up was addressed		p. 5-6

		Case-control study - If applicable, explain how matching of cases and controls was addressed<br>*Cross-sectional study* - If applicable, describe analytical methods taking account of sampling strategy<br>(e) Describe any sensitivity analyses		
Data access and cleaning methods		..	RECORD 12.1: Authors should describe the extent to which the investigators had access to the database population used to create the study population.<br><br>RECORD 12.2: Authors should provide information on the data cleaning methods used in the study.	3-5
Linkage		..	RECORD 12.3: State whether the study included person-level, institutional-level, or other data linkage across two or more databases. The methods of linkage and methods of linkage quality evaluation should be provided.	p. 3-5
**Results**				
Participants	13	(a) Report the numbers of individuals at each stage of the study (*e.g.*, numbers potentially eligible, examined for eligibility, confirmed eligible, included in the study, completing follow-up, and analysed)<br>(b) Give reasons for non-participation at each stage.<br>(c) Consider use of a flow diagram	RECORD 13.1: Describe in detail the selection of the persons included in the study (*i.e.*, study population selection) including filtering based on data quality, data availability and linkage. The selection of included persons can be described in the text and/or by means of the study flow diagram.	p. 6-7
Descriptive data	14	(a) Give characteristics of study participants (*e.g.*, demographic, clinical, social) and information on exposures and potential confounders<br>(b) Indicate the number of participants with missing data for each variable of interest<br>(c) *Cohort study* - summarise follow-up time (*e.g.*, average and total amount)		p. 6

Outcome data	15	*Cohort study* - Report numbers of outcome events or summary measures over time<br>*Case-control study* - Report numbers in each exposure category, or summary measures of exposure<br>*Cross-sectional study* - Report numbers of outcome events or summary measures		p. 6-7
Main results	16	(a) Give unadjusted estimates and, if applicable, confounder-adjusted estimates and their precision (e.g., 95% confidence interval). Make clear which confounders were adjusted for and why they were included<br>(b) Report category boundaries when continuous variables were categorized<br>(c) If relevant, consider translating estimates of relative risk into absolute risk for a meaningful time period		p. 6-7
Other analyses	17	Report other analyses done—e.g., analyses of subgroups and interactions, and sensitivity analyses		p. 6-7
**Discussion**				
Key results	18	Summarise key results with reference to study objectives		p. 7-9
Limitations	19	Discuss limitations of the study, taking into account sources of potential bias or	RECORD 19.1: Discuss the implications of using data that were not created or collected to answer the specific research question(s). Include discussion of	p. 9

		imprecision. Discuss both direction and magnitude of any potential bias	misclassification bias, unmeasured confounding, missing data, and changing eligibility over time, as they pertain to the study being reported.	
Interpretation	20	Give a cautious overall interpretation of results considering objectives, limitations, multiplicity of analyses, results from similar studies, and other relevant evidence		p. 7-10
Generalisability	21	Discuss the generalisability (external validity) of the study results		p. 8-10
**Other Information**				
Funding	22	Give the source of funding and the role of the funders for the present study and, if applicable, for the original study on which the present article is based		p. 1,6,10
Accessibility of protocol, raw data, and programming code		..	RECORD 22.1: Authors should provide information on how to access any supplemental information such as the study protocol, raw data, or programming code.	p. 10

Table 4: Characteristics of individuals in Scotland with first vaccination who were hospitalised or died due to COVID-19 (including events <14 days post-vaccination)

**Characteristic**	**Total vaccination coverage**	**Hospitalisation or death due to COVID-19 <14 days of first vaccination (% of total)**		
		**Both vaccines**	**BNT162b2**	**ChAdOx1**
Total	2,572,655	1843	824	1019
Sex				
Female	1,418,641 (55.1)	1017 (55.2)	473 (57.4)	544 (53.4)
Male	1,154,014 (44.9)	826 (44.8)	351 (42.6)	475 (46.6)
Age group (years)				
18-64	1,659,828 (64.5)	403 (21.9)	183 (22.2)	220 (21.6)
65-79	697,687 (27.1)	509 (27.6)	231 (28.0)	278 (27.3)
>=80	215,140 (8.4)	931 (50.5)	410 (49.8)	521 (51.1)
Prior history of COVID-19				
No	2,488,904 (96.7)	1784 (96.8)	781 (94.8)	1003 (98.4)

Yes	83,751 (3.3)	59 (3.2)	43 (5.2)	16 (1.6)
Prior history of hospitalisation				
No	2,511,840 (97.6)	1627 (88.3)	769 (93.3)	858 (84.2)
Yes	60,815 (2.4)	216 (11.7)	55 (6.7)	161 (15.8)
Elderly care home				
No	2,553,387 (99.3)	1518 (82.4)	504 (61.2)	1014 (99.5)
Yes	19,268 (0.7)	325 (17.6)	320 (38.8)	5 (0.5)
Deprivation status †				
1 – Most deprived	469,021 (18.2)	514 (27.9)	206 (25.0)	308 (30.2)
2	504,965 (19.6)	440 (23.9)	186 (22.6)	254 (24.9)
3	526,160 (20.5)	298 (16.2)	157 (19.1)	141 (13.8)
4	537,760 (20.9)	291 (15.8)	148 (18.0)	143 (14.0)
5 – Least deprived	521,313 (20.3)	281 (15.2)	111 (13.5)	170 (16.7)
Unknown	13,436 (0.5)	19 (1.0)	16 (1.9)	3 (0.3)

Urban/rural score				
1 – Large urban area	776,279 (30.3)	594 (32.6)	232 (28.7)	362 (35.6)
2	991,272 (38.7)	733 (42.4)	340 (42.1)	433 (42.6)
3	258,072 (10.1)	168 (9.2)	65 (8.0)	103 (10.1)
4	138,908 (5.4)	75 (4.1)	47 (5.8)	28 (2.8)
5	248,265 (9.7)	171 (9.4)	101 (12.5)	70 (6.9)
6 – Remote rural area	146,423 (5.7)	43 (2.4)	23 (2.8)	20 (2.0)
Unknown	13,436 (0.5)	19 (1.0)	16 (1.9)	3 (0.3)
Smoking status				
Ex-smoker	432,946 (16.8)	466 (25.3)	182 (22.1)	284 (27.9)
Non-smoker	999,744 (38.9)	612 (33.2)	266 (32.3)	346 (34.0)
Smoker	621,735 (24.2)	477 (25.9)	182 (22.1)	295 (28.9)
Unknown	518,230 (20.1)	288 (15.6)	194 (23.5)	94 (9.2)
Number of comorbidities				

0	1,233,347 (47.9)	300 (16.3)	130 (15.8)	170 (16.7)
1	760,383 (29.6)	347 (18.8)	134 (16.3)	213 (20.9)
2	338,082 (13.1)	383 (20.8)	166 (20.1)	217 (21.3)
3	141,296 (5.5)	332 (18.0)	158 (19.2)	174 (17.1)
4	60,233 (2.3)	215 (11.7)	104 (12.6)	111 (10.9)
≥5	39,314 (1.5)	266 (14.4)	132 (16.0)	134 (13.2)
Number of previous tests §				
0	2,059,974 (80.1)	885 (48.0)	240 (29.1)	645 (63.3)
1	313,118 (12.2)	272 (14.8)	126 (15.3)	146 (14.3)
2	78,277 (3.0)	157 (8.5)	84 (10.2)	73 (7.2)
3	29,445 (1.1)	134 (7.3)	99 (12.0)	35 (3.4)
4-9	47,525 (1.8)	285 (15.5)	193 (23.4)	92 (9.0)
10+	44,316 (1.7)	110 (6.0)	82 (10.0)	28 (2.7)
Asthma	352,773 (13.7)	270 (14.7)	86 (10.4)	184 (18.1)

Chronic kidney disease (stages 3-5)*	152,138 (5.9)	487 (26.4)	189 (22.9)	298 (29.2)
Liver cirrhosis	20,307 (0.8)	22 (1.2)	6 (0.7)	16 (1.6)
Chronic neurological condition	16,903 (0.3)	15 (0.8)	8 (1.0)	7 (0.7)
Heart failure	44,710 (1.7)	141 (7.7)	44 (5.3)	97 (9.5)
Diabetes (type 1)	20,331 (0.8)	14 (0.8)	5 (0.6)	9 (0.9)
Diabetes (type 2)	244,070 (9.5)	440 (23.9)	169 (20.5)	271 (26.6)
Dementia	34,642 (1.3)	349 (18.9)	280 (34.0)	69 (6.8)
Coronary heart disease	190,141 (7.4)	404 (21.9)	151 (18.3)	253 (24.8)
*https://www.nice.org.uk/guidance/cg182/chapter/introduction#kidney-disease-improving-global-outcomes-gfr-categories				

Table 5: Association from univariate Poisson model for demographic and clinical characteristics of patients with hospitalisation or death due to COVID-19 illness following first vaccination dose

Characteristics	Unadjusted rate ratios (95% CI)		
	**Both vaccines**	**BNT162b2**	**ChAdOx1**
Time since first vaccination			
14-20 days	1.0	1.0	1.0
21-27 days	0.70 (0.60-0.83)	0.72 (0.56-0.93)	0.68 (0.55-0.84)
28-34 days	0.57 (0.47-0.68)	0.65 (0.50-0.84)	0.48 (0.36-0.62)
35-41days	0.47 (0.39-0.58)	0.44 (0.33-0.59)	0.48 (0.36-0.63)
42-128 days	0.37 (0.32-0.43)	0.37 (0.30-0.46)	0.34 (0.27-0.42)
Sex			
Female	1.0	1.0	1.0
Male	1.04 (0.93-1.17)	1.18 (1.00-1.39)	0.97 (0.83-1.14)
Age group (years)			
18-64	1.0	1.0	1.0
65-79	1.78 (1.50-2.10)	1.89 (1.48-2.42)	1.68 (1.34-2.12)
≥80	10.03 (8.65-11.67)	35.47 (28.63-44.31)	5.49 (4.48-6.77)
Prior history of COVID-19*			
No	1.0	1.0	1.0
Yes	1.18 (0.86-1.59)	1.35 (0.92-1.90)	0.81 (0.42-1.39)
Prior history of hospitalisation			
No	1.0	1.0	1.0
Yes	4.83 (4.01-5.78)	5.09 (3.65-6.89)	5.15 (4.08-6.42)
Elderly care home			
No	1.0	1.0	1.0
Yes	26.53 (23.08-30.38)	32.28 (27.42-37.95)	9.14 (2.27-23.79)

Deprivation status †			
1 – Most deprived	2.00 (1.67-2.40)	1.77 (1.36-2.32)	2.22 (1.73-2.85)
2	1.59 (1.32-1.92)	1.52 (1.16-2.00)	1.66 (1.29-2.16)
3	1.05 (0.86-1.28)	1.34 (1.02-1.78)	0.80 (0.59-1.08)
4	1.05 (0.85-1.28)	1.16 (0.87-1.54)	0.94 (0.70-1.26)
5 – Least deprived	1.0	1.0	1.0
Urban/rural index			
1 (Large urban areas, Other urban areas, Accessible small towns)	1.0	1.0	1.0
2 (Remote small towns, Accessible rural area, Remote rural area)	0.78 (0.67-0.91)	1.22 (1.00-1.48)	0.48 (0.38-0.61)
Smoking status			
Non-smoker	1.0	1.0	1.0
Smoker	1.22 (1.05-1.42)	1.16 (0.93-1.45)	1.28 (1.04-1.56)
Ex-smoker	1.61 (1.38-1.86)	1.72 (1.38-2.13)	1.55 (1.27-1.90)
Unknown	1.07 (0.90-1.27)	1.43 (1.14-1.78)	0.66 (0.49-0.88)
Number of risk groups ‡			
0	1.0	1.0	1.0
1	1.64 (1.35-2.01)	1.76 (1.30-2.39)	1.53 (1.17-2.01)
2	3.99 (3.30-4.84)	5.95 (4.51-7.89)	2.94 (2.26-3.83)
3	7.89 (6.50-9.60)	14.23 (10.79-18.87)	5.04 (3.84-6.64)
4	10.23 (8.18-12.78)	20.28 (14.82-27.71)	6.06 (4.38-8.32)
≥ 5	19.85 (16.13-24.43)	40.71 (30.47-54.55)	11.30 (8.36-15.23)
Number of previous tests §			
0	1.0	1.0	1.0

1	2.26 (1.90-2.66)	3.04 (2.33-3.94)	1.95 (1.90-2.66)
2	4.92 (3.98-6.01)	7.92 (5.91-10.51)	3.41 (2.45-4.62)
3	10.97 (8.82-13.50)	21.90 (16.67-28.57)	3.99 (2.43-6.14)
4-9	12.80 (10.88-15.01)	20.05 (16.02-25.10)	8.13 (6.05-10.71)
10+	4.53 (3.51-5.76)	5.23 (3.78-7.11)	8.47 (5.16-13.04)

* Unadjusted rate ratio for prior history of SARS-CoV-2 infection couldn't be estimated for individuals vaccinated with ChAdOx1 vaccine due to no hospitalisation or death due COVID-19 among them

* Prior history of hospitalisation status defined as a admission to hospital within 4 weeks prior to 1st dose vaccination

† Deprivation status: Scottish Index of Multiple Deprivation (SIMD) 2020

‡ Number of risk groups: Individual QCOVID risk groups found in supplementary table 7

§ Number of previous tests: Proxy for working in a high-risk occupation (e.g. healthcare worker)

Table 6: Associations from multivariate Poisson models for demographic and clinical characteristics of patients with hospitalisation or death due to COVID-19 illness (U07.1 coding only) following first vaccination dose

Characteristics	Adjusted rate ratios (95% CI)		
	Both vaccines	BNT162b2	ChAdOx1
Time since first vaccination			
14-20 days	1.0	1.0	1.0
21-27 days	0.61 (0.49, 0.75)	0.57 (0.39, 0.82)	0.63 (0.49, 0.81)
28-34 days	0.30 (0.23, 0.39)	0.27 (0.16, 0.43)	0.31 (0.23, 0.44)
35-41days	0.27 (0.20, 0.36)	0.27 (0.17, 0.44)	0.27 (0.19, 0.39)
42-128 days	0.11 (0.08, 0.14)	0.16 (0.11, 0.23)	0.08 (0.06, 0.11)
Sex			
Female	1.0	1.0	1.0
Male	1.26 (1.06, 1.49)	1.37 (1.03, 1.82)	1.17 (0.95, 1.43)
Age group (years)			
18-64	1.0	1.0	1.0
65-79	1.66 (1.31, 2.10)	2.26 (1.49, 3.41)	0.93 (0.65, 1.32)
≥80	3.19 (2.50, 4.06)	3.45 (2.17, 5.47)	1.26 (0.83, 1.92)
Prior history of COVID-19*			
No	1.0	1.0	1.0
Yes	0.00 (0.00, Inf)	0.00 (0.00, Inf)	0.00 (0.00, Inf)
Prior history of hospitalisation			
No	1.0	1.0	1.0
Yes	3.16 (2.42, 4.14)	2.71 (1.49, 4.94)	3.23 (2.38, 4.39)
Elderly care home			
No	1.0	1.0	1.0

Yes	1.00 (0.72, 1.40)	1.03 (0.68, 1.58)	0.90 (0.12, 6.62)
Deprivation status †			
1 – Most deprived	0.61 (0.47, 0.80)	0.72 (0.48, 1.10)	0.55 (0.38, 0.78)
2	0.74 (0.58, 0.96)	0.69 (0.45, 1.06)	0.79 (0.57, 1.09)
3	0.57 (0.44, 0.75)	0.59 (0.36, 0.94)	0.56 (0.40, 0.78)
4	1.03 (0.83, 1.29)	0.91 (0.63, 1.33)	1.09 (0.82, 1.44)
5 – Least deprived	1.0	1.0	1.0
Urban/rural index			
1 (Large urban areas, Other urban areas, Accessible small towns)	1.0	1.0	1.0
2 (Remote small towns, Accessible rural area, Remote rural area)	1.08 (0.83, 1.40)	0.98 (0.64, 1.50)	1.13 (0.82, 1.57)
Smoking status			
Non-smoker	1.0	1.0	1.0
Smoker	1.14 (0.92, 1.41)	1.28 (0.90, 1.84)	1.04 (0.79, 1.36)
Ex-smoker	1.24 (1.00, 1.54)	1.28 (0.88, 1.85)	1.21 (0.93, 1.57)
Unknown	0.97 (0.72, 1.29)	0.77 (0.50, 1.18)	1.25 (0.83, 1.88)
Number of risk groups ‡			
0	1.0	1.0	1.0
1	1.37 (1.03, 1.81)	1.55 (1.00, 2.41)	1.15 (0.80, 1.65)
2	2.48 (1.88, 3.28)	3.00 (1.91, 4.71)	1.91 (1.34, 2.72)
3	2.92 (2.15, 3.96)	3.49 (2.10, 5.83)	2.19 (1.50, 3.22)
4	2.79 (1.95, 3.99)	1.80 (0.89, 3.66)	2.71 (1.78, 4.14)

≥5	4.98 (3.57, 6.93)	5.17 (2.95, 9.05)	4.04 (2.67, 6.10)
Number of previous tests §			
0	1.0	1.0	1.0
1	1.69 (1.33, 2.15)	2.01 (1.30, 3.11)	1.65 (1.23, 2.20)
2	2.29 (1.68, 3.14)	2.99 (1.79, 4.99)	2.12 (1.40, 3.20)
3	2.45 (1.66, 3.60)	3.44 (1.95, 6.07)	2.02 (1.12, 3.63)
4-9	3.25 (2.46, 4.29)	4.54 (2.91, 7.11)	2.86 (1.91, 4.27)
10+	1.69 (1.11, 2.57)	1.91 (1.07, 3.41)	2.76 (1.39, 5.46)

* Adjusted rate ratio for prior history of SARS-CoV-2 infection couldn't be estimated for individuals vaccinated with ChAdOx1 vaccine due to no hospitalisation or death due COVID-19 among them
† Deprivation status: Scottish Index of Multiple Deprivation (SIMD) 2020
‡ Number of risk groups: Individual QCOVID risk groups found in supplementary table 7
§ Number of previous tests: Proxy for working in a high-risk occupation (e.g. healthcare worker)

Table 7: QCovid risk groups and codes

QCOVID risk group	Code
Atrial fibrillation	Q_DIAG_AF
Asthma	Q_DIAG_ASTHMA
Blood cancer	Q_DIAG_BLOOD_CANCER
Heart failure	Q_DIAG_CCF
Cerebral palsy	Q_DIAG_CEREBALPALSY
Coronary heart disease	Q_DIAG_CHD
Cirrhosis	Q_DIAG_CIRRHOSIS
Congenital heart disease	Q_DIAG_CONGEN_HD
COPD	Q_DIAG_COPD
Dementia	Q_DIAG_DEMENTIA
Diabetes type 1	Q_DIAG_DIABETES_1
Diabetes type 2	Q_DIAG_DIABETES_2
Epilepsy	Q_DIAG_EPILEPSY
Fracture	Q_DIAG_FRACTURE
Neurological disorder	Q_DIAG_NEURO
Parkinson’s	Q_DIAG_PARKINSONS
Pulmonary hypertension	Q_DIAG_PULM_HYPER
Pulmonary rare	Q_DIAG_PULM_RARE
Peripheral vascular disease	Q_DIAG_PVD
Rheumatoid arthritis or SLE	Q_DIAG_RA_SLE

Respiratory cancer	Q_DIAG_RESP_CANCER
Severe mental illness	Q_DIAG_MENT_ILL
Sickle cell disease	Q_DIAG_SICKLE_CELL
Stroke/TIA	Q_DIAG_STROKE
Thrombosis or pulmonary embolus	Q_DIAG_VTE
Care housing category	Q_HOME_CAT
Learning disability or Down's	Q_LEARN_CAT
Kidney disease	Q_DIAG_CKD_LEVEL
More information on codes: https://github.com/EAVE-II/EAVE-II-data-dictionary<br>Ref: Clift, A.K., et al. Living risk prediction algorithm (QCOVID) for risk of hospital admission and mortality from coronavirus 19 in adults: national derivation and validation cohort study. BMJ 371, m3731 (2020).	

Figure 1: Severe COVID-19 outcomes by age and vaccine type (AZ – Oxford-AstraZeneca, PB: Pfizer-BioNTech) (A) hospitalisation or death combined (B) hospitalisation (C) deaths. The x-axis represents age groups and y-axis represents the number of incidents.

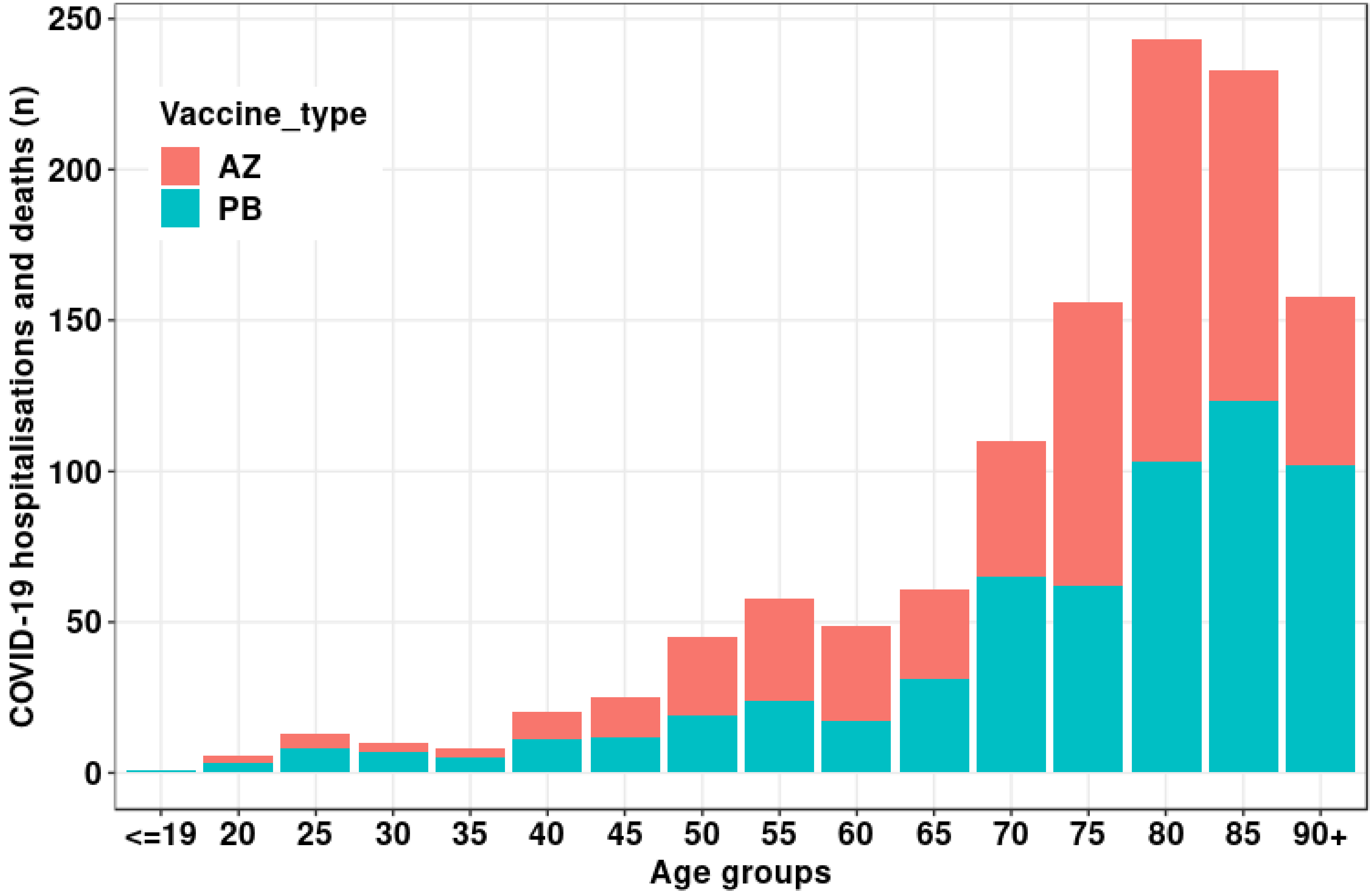

A. **Hospitalisation or death due to COVID-19 by age groups**

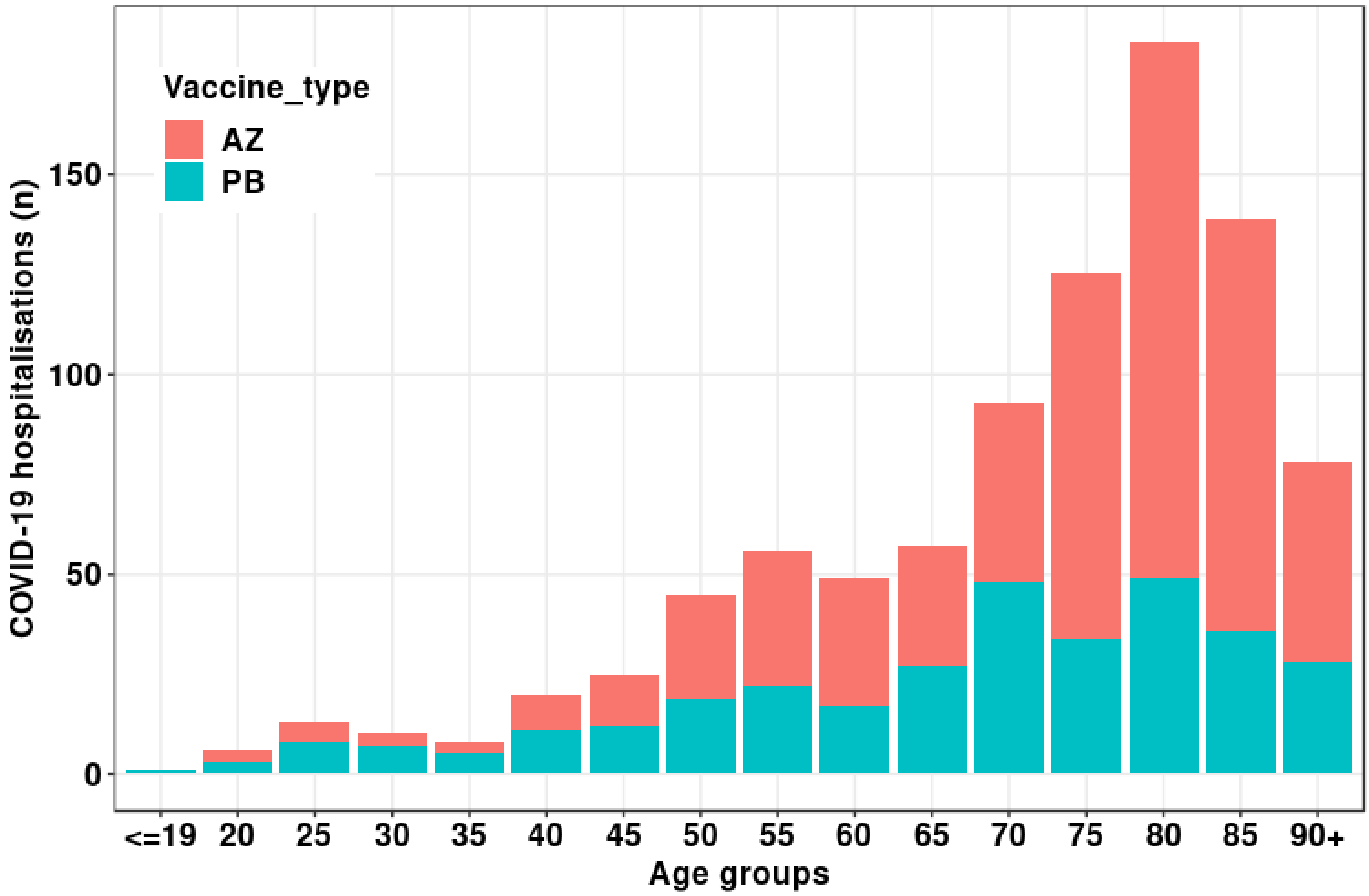

B. **Hospitalisation due to COVID-19 by age groups**

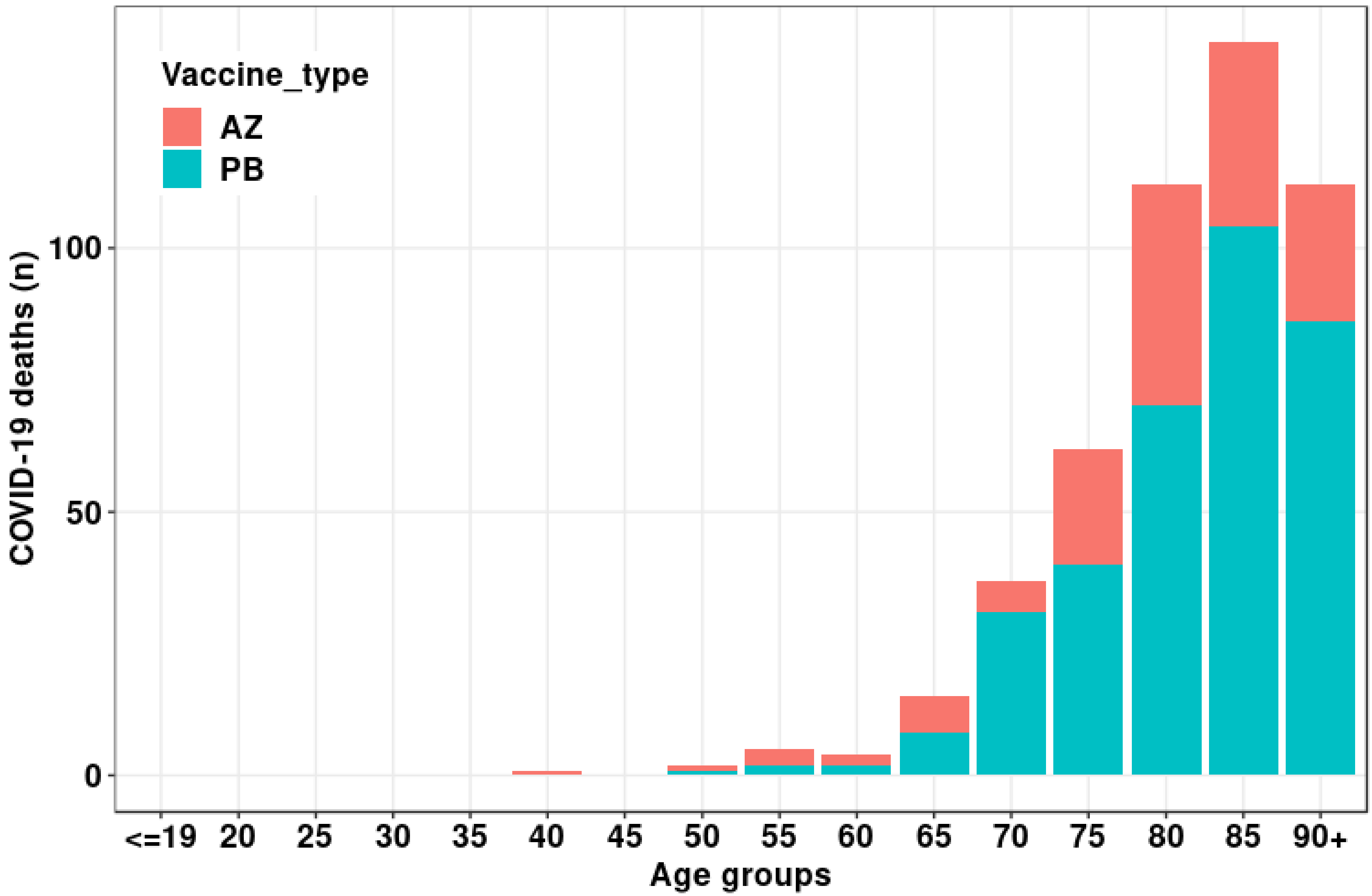

C. **Deaths due to COVID-19 by age groups**

Figure 2: Plots comparing the proportion of individuals vaccinated and proportion of individuals with events by (A) age groups, (B) number of different conditions (QCovid Risk Groups), and (C) socioeconomic status measured by SIMD (1=most deprived, 5=least deprived)

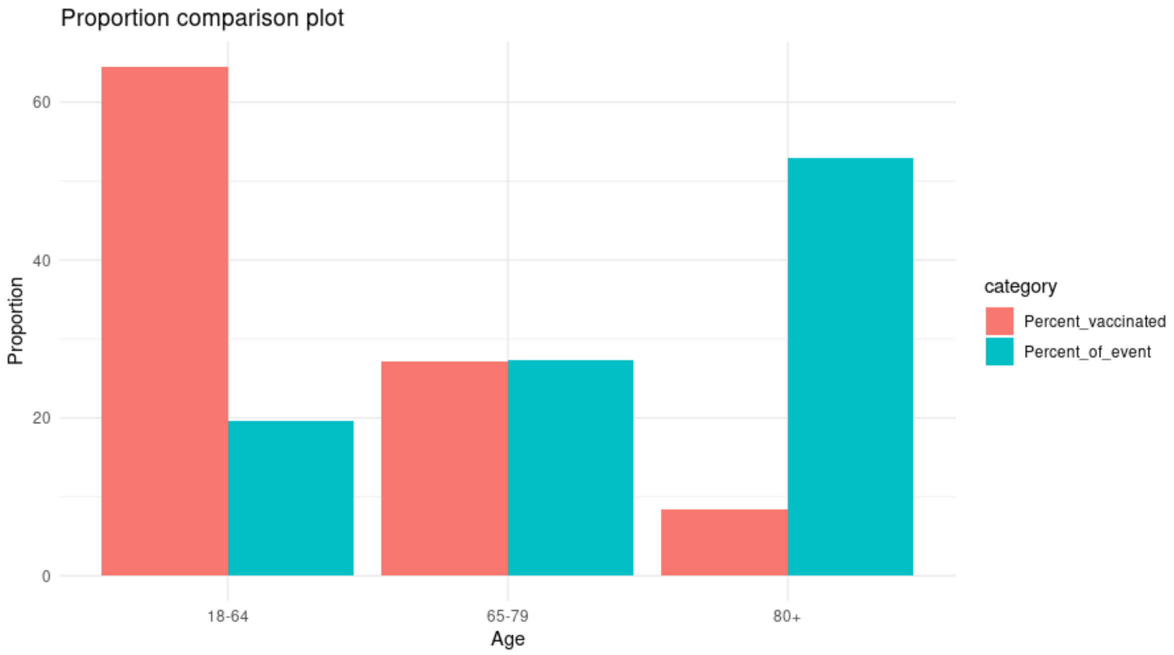

A. **Proportion of individuals vaccinated and proportion of individuals with events by age group**

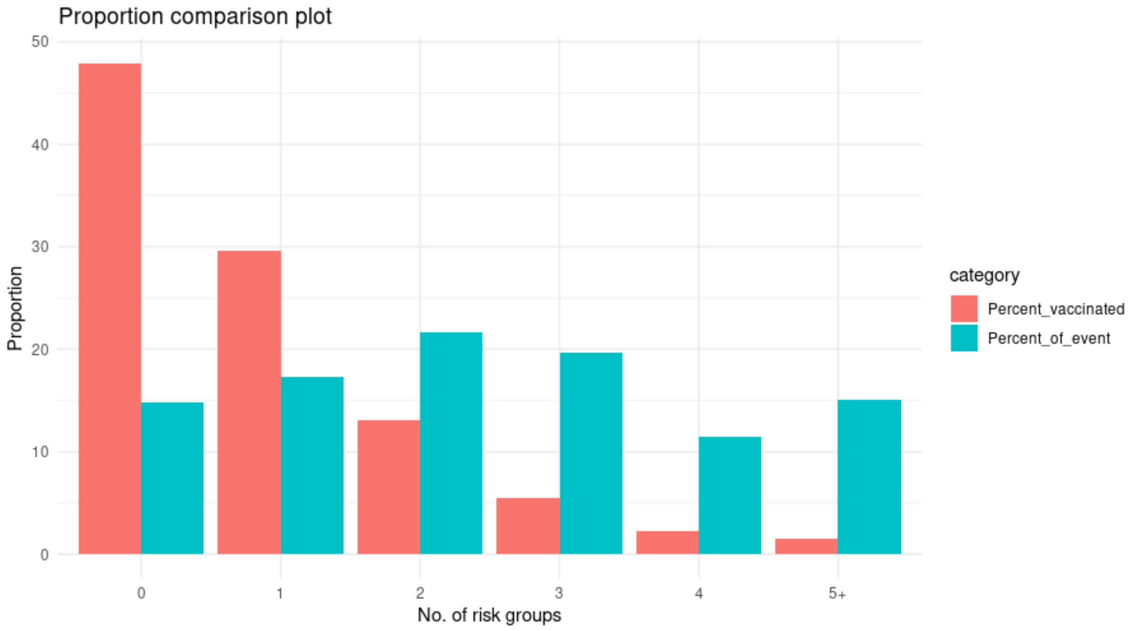

B. **Proportion of individuals vaccinated and proportion of individuals with events by number of different conditions (QCovid Risk Groups)**

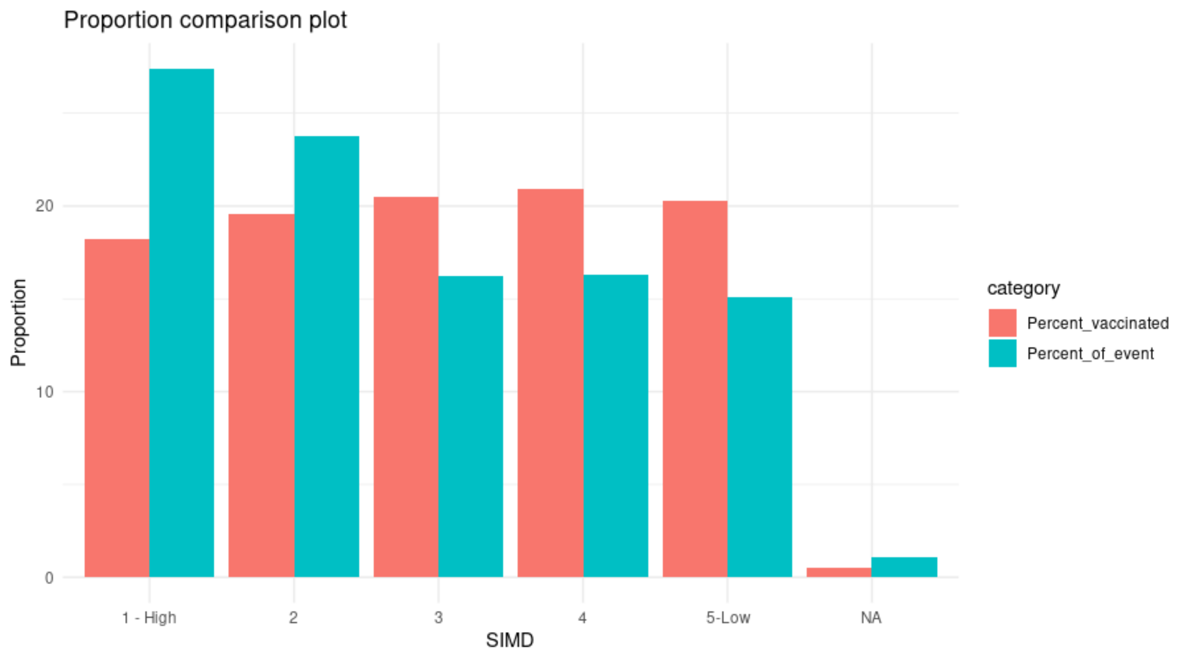

C. **Proportion of individuals vaccinated and proportion of individuals with events by socioeconomic status measured by SIMD (1=most deprived, 5=least deprived)**

Figure 3: Cumulative incidence of hospitalisation or death due to COVID-19 for vaccine type (A), age groups (B), number of risk groups (C) and socio-economic status (D). X-axis represents number of days post-vaccination and y-axis represents the cumulative incidence*

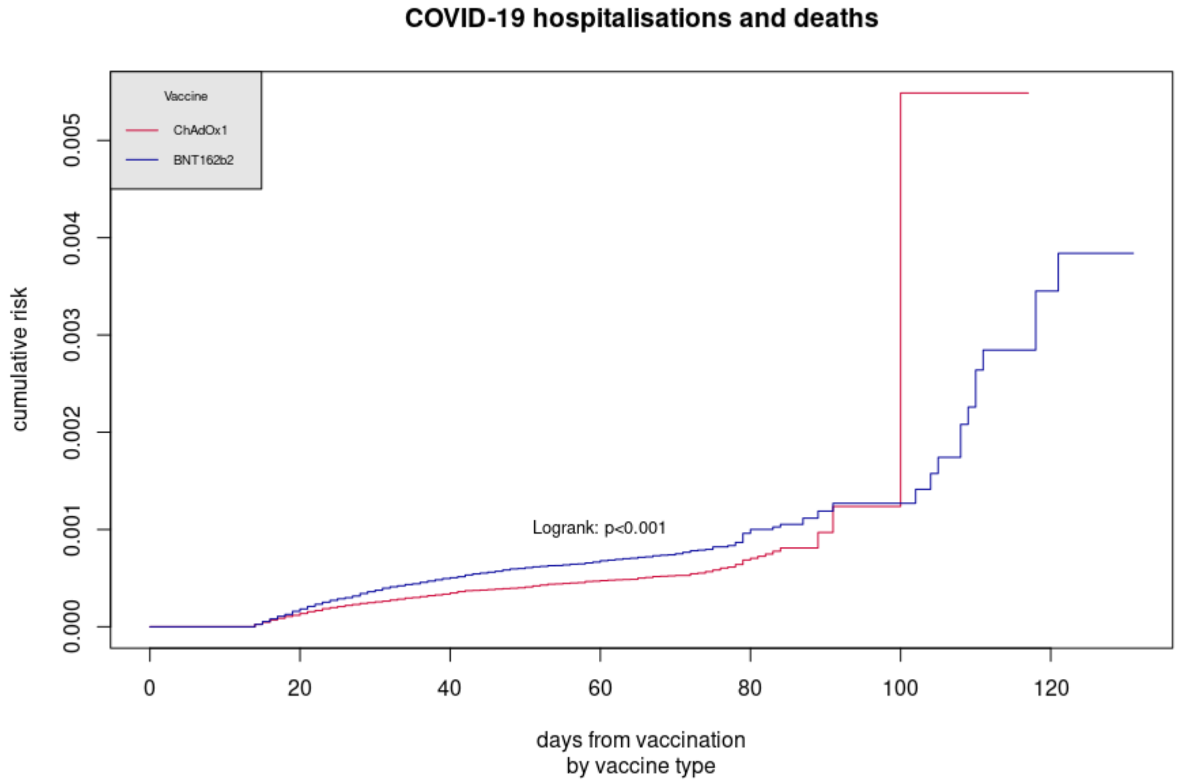

A. Cumulative risk by vaccine type

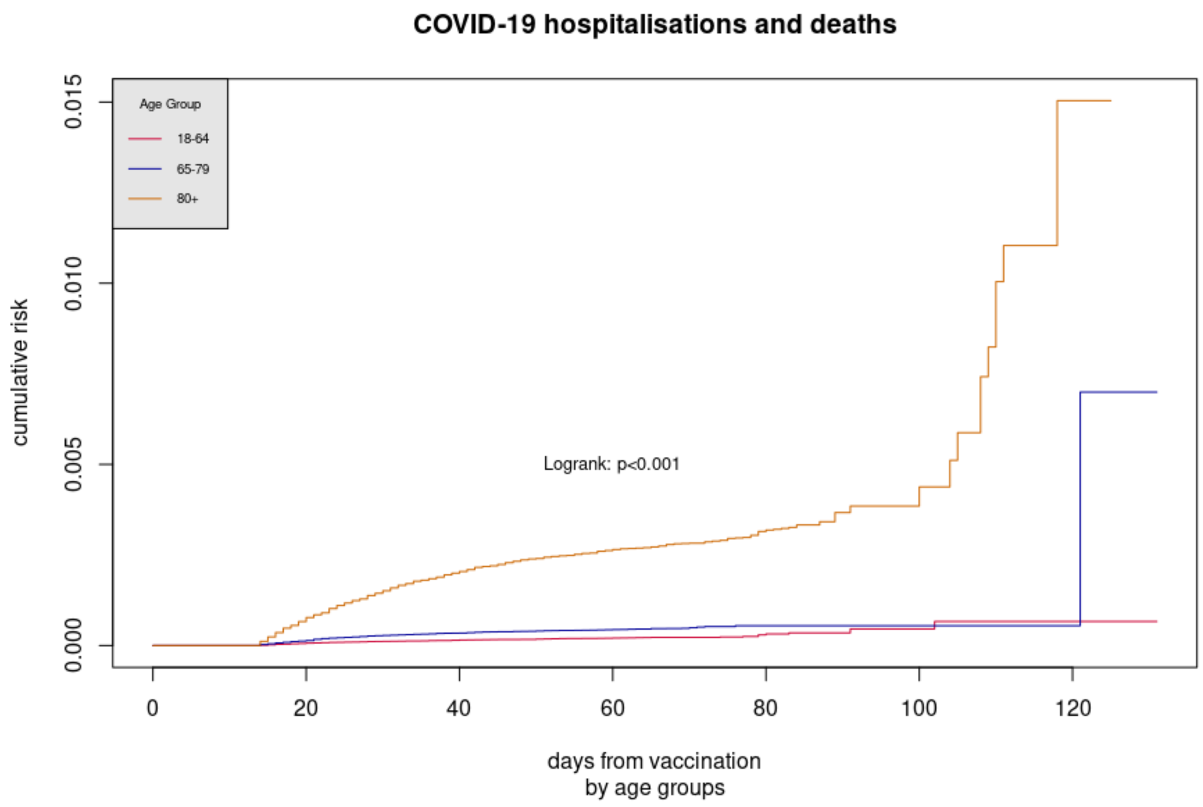

B. Cumulative risk by age groups

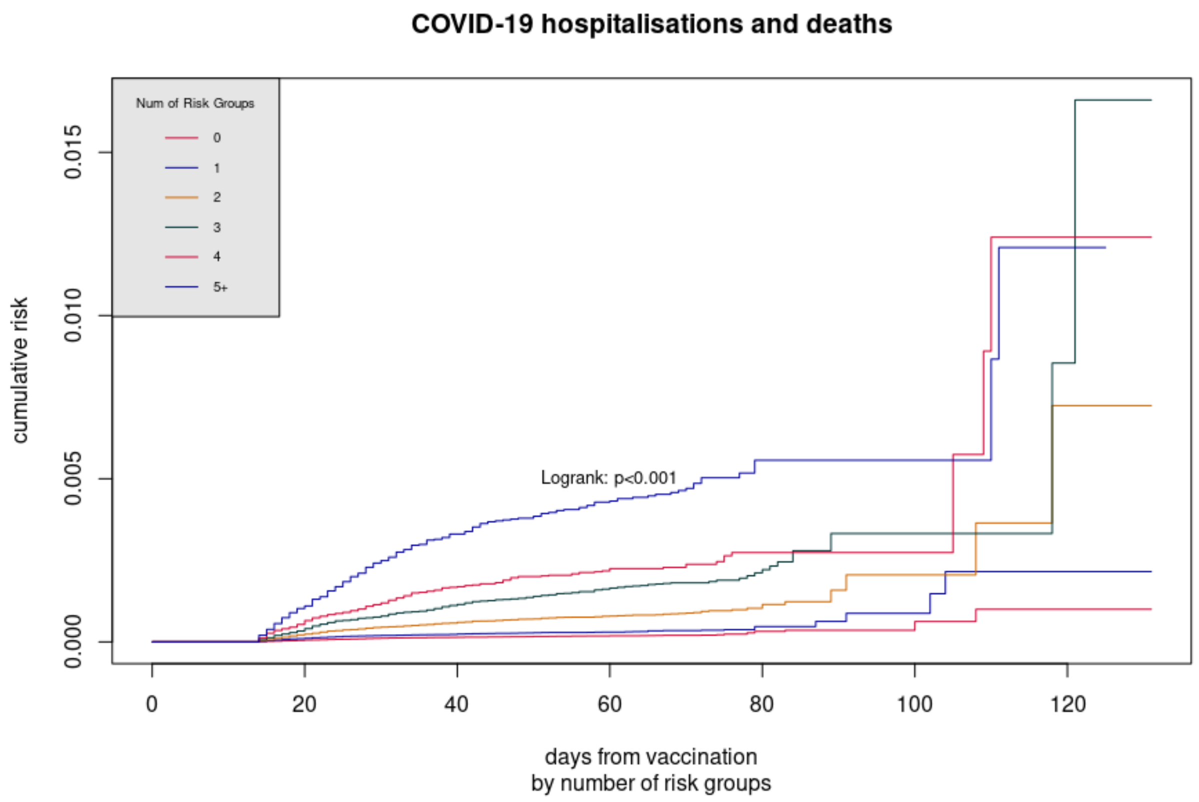

C. Cumulative risk by number of risk groups

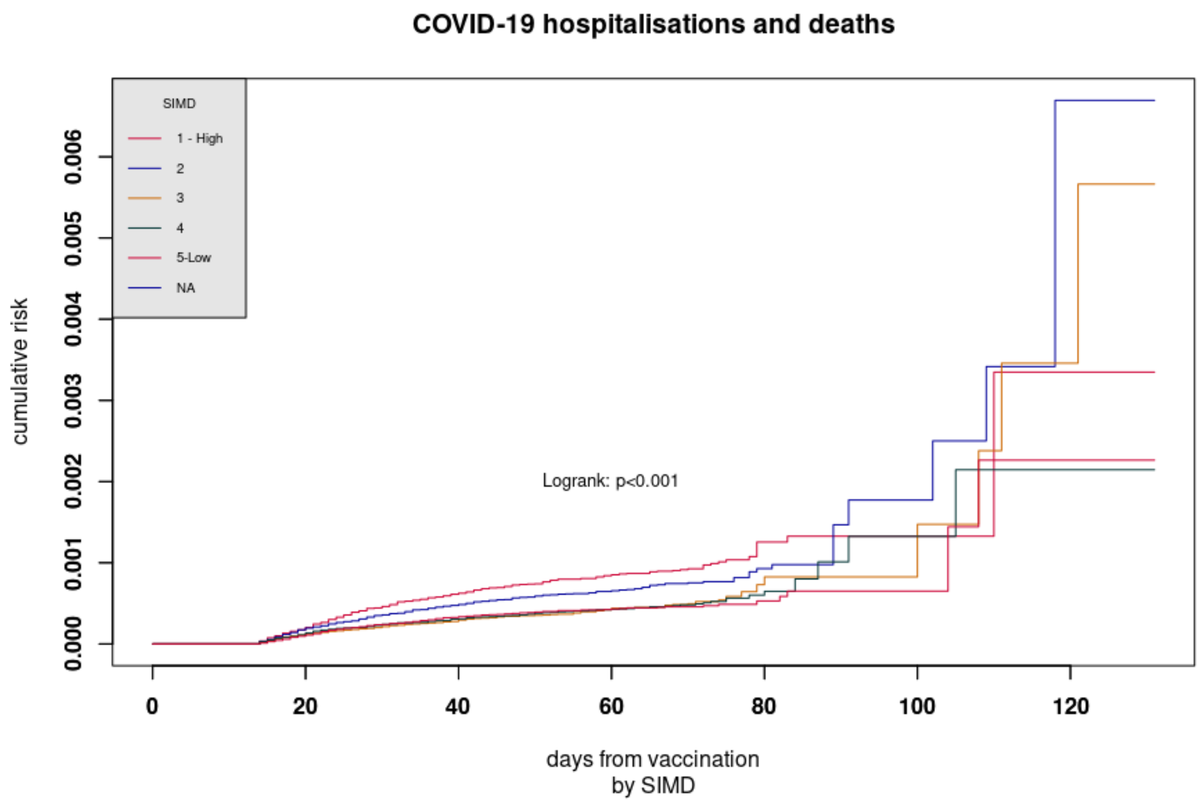

D. Cumulative risk by deprivation status

(1 - High represents most deprived group and 5 - Low represents least deprived group)

*Please note the y-axis scale differs between figures which are reported unadjusted

	20	40	60	80	100	120
Numbers at risk	2,411,612	2,266,813	1,661,549	1,065,542	95,999	7,613

Censored	144,438	604,755	595,792	969,456	88,374	7,601
Events	361	509	215	87	12	12
